# Author Correction: Increased burden of ultra-rare structural variants localizing to boundaries of topologically associated domains in schizophrenia

**DOI:** 10.1038/s41467-021-27826-z

**Published:** 2022-01-05

**Authors:** Matthew Halvorsen, Ruth Huh, Nikolay Oskolkov, Jia Wen, Sergiu Netotea, Paola Giusti-Rodriguez, Robert Karlsson, Julien Bryois, Björn Nystedt, Adam Ameur, Anna K. Kähler, NaEshia Ancalade, Martilias Farrell, James J. Crowley, Yun Li, Patrik K. E. Magnusson, Ulf Gyllensten, Christina M. Hultman, Patrick F. Sullivan, Jin P. Szatkiewicz

**Affiliations:** 1 Department of Genetics, University of North Carolina, Chapel Hill, NC 27599 USA; 2 Department of Biostatistics, University of North Carolina, Chapel Hill, NC 27599 USA; 3 Department of Biology, National Bioinformatics Infrastructure Sweden, Science for Life Laboratory, Lund University, 22362 Lund, Sweden; 4 Department of Biology and Biological Engineering, National Bioinformatics Infrastructure Sweden, Science for Life Laboratory, Chalmers University of Technology, 41258 Göteborg, Sweden; 5 Department of Medical Epidemiology and Biostatistics, Karolinska Institutet, 17177 Stockholm, Sweden; 6 Department of Cell and Molecular Biology, National Bioinformatics Infrastructure Sweden, Science for Life Laboratory, Uppsala University, 75237 Uppsala, Sweden; 7 Department of Immunology, Genetics and Pathology, Science for Life Laboratory, Uppsala University, 75185 Uppsala, Sweden; 8 Department of Psychiatry, University of North Carolina, Chapel Hill, NC 27599 USA; 9 Department of Clinical Neuroscience, Karolinska Institutet, 17177 Stockholm, Sweden

**Keywords:** DNA sequencing, Next-generation sequencing, Genetics of the nervous system, Schizophrenia

Correction to: *Nature Communications* 10.1038/s41467-020-15707-w, published online 15 April 2020.

In this article, the grant number NIMH K01 MH109772 relating to the National Institute of Mental Health for Paola Giusti-Rodriguez was omitted. The original article has been corrected.

