# Supplementary material for: Increased burden of ultra-rare structural variants localizing to boundaries of topologically associated domains in schizophrenia
Source: Nat Commun. 2020 Apr 15;11:1842. doi: 10.1038/s41467-020-15707-w (PMC7160146; doi:10.1038/s41467-020-15707-w)
Supplement: Supplementary file 1 — Supplementary Information [file 41467_2020_15707_MOESM1_ESM.pdf]

Supplementary Information

Increased burden of ultra-rare structural variants localizing to boundaries of topologically associated do mains in schizophrenia

Halvorsen [#], Huh [#], Oskolkov [#], Wen [#], et al.

Table of Content

Table of Content 2

Supplementary Tables 4

Supplementary Table 1 Published studies that performed WGS in schizophrenia samples 4
Supplementary Table 2: Genotype concordance of SNV/Indels detected from WGS 5
Supplementary Table 2a: Overall genotype concordance 5
Supplementary Table 2b: Genotype concordance for common variants (MAF > 0.05) 5
Supplementary Table 2c: Genotype concordance for low-frequency variants (MAF 0.005-0.05) 5
Supplementary Table 2d: Genotype concordance for rare variants (MAF < 0.005) but not singleton 5
Supplementary Table 2e: Genotype concordance for singletons (Minor Allele Count =1) 5
Supplementary Table 3: Genotype concordance of DEL and DUP detected from WGS 6
Supplementary Table 4: Summary statistics for repeat expansions detected from WGS 7
Supplementary Table 5: Results of burden analysis for ultra-rare SNV/Indels across annotations 8
Supplementary Table 6: Genome-wide burden of rare deletions and duplications – Confirmation of prior findings 10
Supplementary Table 7: Results of burden analysis of ultra-rare SVs: genome-wide burden 11
Supplementary Table 8: Results of burden analysis for ultra-rare SVs: across annotations 12
Supplementary Table 9: Burden of ultra-rare TADs-affecting SVs – by overlapping criterion 13
Supplementary Table 10 Burden of ultra-rare TADs-affecting SVs– by variant type 13
Supplementary Table 11: Size distribution of ultra-rare TADs-affecting SVs in schizophrenia cases 13
Supplementary Table 12 Genomic features nearby ultra-rare TADs-affecting SVs in schizophrenia cases 13

Supplementary Figures 14

Supplementary Figure 1: Distribution of mean depth of coverage 14
Supplementary Figure 2: Percentage of bases covered versus depth of coverage 15
Supplementary Figure 3: Ancestry principal component plots 16
Supplementary Figure 3a. WGS samples in global context 16
Supplementary Figure 3b. WGS samples in European context 17
Supplementary Figure 3c. WGS samples in Swedish context 17
Supplementary Figure 4: Size distribution of DEL, DUP, INV 18
Supplementary Figure 5: Size distribution of MEI 19
Supplementary Figure 6: Power calculation for association analysis 20
Supplementary Figure 7: Density plots of ultra-rare SNV/indel counts before and after outlier pruning 21
Supplementary Figure 8: URV outlier status and principal components 22
Supplementary Figure 9: Burden of coding URV in LoF-intolerant genes overlapping old and new Agilent kit loci 23
Supplementary Figure 10: Burden of noncoding URVs across binned regions by sequence constraint 24
Supplementary Figure 11: Burden of noncoding URVs in constrained nucleotides in brain functional annotations 25
Supplementary Figure 12: Genome-wide burden of ultra-rare SVs 26
Supplementary Figure 13: Distribution of coding versus noncoding ultra-rare SVs 27
Supplementary Figure 14: Burden of ultra-rare TADs-affecting SVs – by overlap criterion 28
Supplementary Figure 15: Burden of ultra-rare TADs-affecting SVs – by variant type 29
Supplementary Figure 16: IGV plot for a deletion that overlapped TAD boundaries and was confirmed by GWA SNP array 30
Supplementary Figure 17: IGV plot for a duplication that overlapped TAD boundaries and was confirmed by GWA SNP array 31
Supplementary Figure 18: IGV plot for a deletion that overlapped TAD boundaries and was not found by GWA SNP array 32
Supplementary Figure 19: IGV plot for a duplication that overlapped TAD boundaries and was not found by GWA SNP array 33
Supplementary Figure 20: QQ and Manhattan plots for common SNVs and indels 34
Supplementary Figure 21: QQ and Manhattan plots for common DEL 35
Supplementary Figure 22: QQ and Manhattan plots for common DUP 36

Supplementary Figure 23: QQ and Manhattan plots for common INV 37
Supplementary Figure 24: QQ and Manhattan plots for common ALU 38
Supplementary Figure 25: QQ and Manhattan plots for common LINE1 39
Supplementary Figure 26: QQ and Manhattan plots for common SVA 40
Supplementary Figure 27: Heritability estimation using WGS 41

Supplementary References 42

Abbreviations:

WGS: whole genome sequencing

WES: whole exome sequencing

GWA: genome-wide association

SNV: single nucleotide variant

SV: structural variant/structural variation

DEL: deletion

DUP: duplication

INV: inversion

MEI: mobile element insertion

URV: ultra-rare variants (SNV/Indels)

LoF: loss of function

Supplementary Tables

Supplementary Table 1 Published studies that performed WGS in schizophrenia samples

First Author	PMID	Ref	Study Design	Sample Size	DNA	Aims
Reay	30532020	[1]	Schizophrenia cases and controls	321 schizophrenia cases, 148 controls	Blood	To evaluate rare variation in retinoid signaling pathway
Merico	26384369	[2]	22q11del carriers with extreme phenotypes	9 individuals with 22q11.2del, 6 with schizophrenia	Blood	To test for rare variation that act with *DGCR*8 haploinsufficiency to confer risk
Khan	29486958	[3]	Multiplex schizophrenia families	300 individuals from 91 families (109 schizophrenia or schizoaffective disorder, 82 other neuropsychiatric diagnoses, 109 unaffected)	Blood	To evaluate smaller copy number variants
Tang	28645778	[4]	Monozygotic twin pairs discordant for schizophrenia	8 twin pairs	Blood	To assess *de novo* mutations or inherited rare variants
Castellani	2918159	[5]	Monozygotic twin pairs discordant for schizophrenia	2 twin pairs and 1 set of parents	Blood or Buccal swabs	To assess *de novo* mutations or inherited rare variants
Homann	27001614	[6]	Multiplex families with psychosis	90 individuals across 9 families with psychosis	Blood or cell lines	To search for rare highly penetrant mutations that segregate with illness
Steinberg	28628109	[7]	Multiplex families with psychosis	10 individuals with psychosis from an Icelandic kindred	Blood	To identify rare highly penetrant mutations
Chen	31481703	[8]	Family based. Each family had at least two affected siblings diagnosed with schizophrenia, at least 1 unaffected sibling, and at least 1 parent.	99 individuals from 20 families of Taiwanese Han Chinese	Lymphoblastoid cell lines transformed from whole blood	To identify functional variants associated with schizophrenia
Bundo	24389010	[9]	Schizophrenia cases and controls	3 cases and 3 controls	Brain and liver	To identify brain-specific L1 transposition
Zhu	bioRxiv doi: 10.1101/660779	[10]	Schizophrenia cases and controls	2 cases and 2 controls	Brain	To identify somatic mobile element insertions

Supplementary Table 2: Genotype concordance of SNV/Indels detected from WGS

We evaluated genotype concordance by computing genotype agreement between whole exome sequencing (WES) [11,12] and WGS. We examined 517,888 SNVs and 28,654 indels in 1,154 of the 1,162 schizophrenia cases across chr1-chr22 that are common between WES and WGS. We evaluated genotype concordance overall and then stratified by MAF categories.

Supplementary Table 2a: Overall genotype concordance

WGS compared to WES	SNVs		Indels	
	Total # comparisons	Concordance rate	Total # comparisons	Concordance rate
Homozygous reference	498,084	0.9999	27,012	0.9978
Heterozygous	397,208	0.9989	20,677	0.9835
Homozygous alternative	79,133	0.9971	3,842	0.9835

Supplementary Table 2b: Genotype concordance for common variants (MAF > 0.05)

WGS compared to WES	SNVs		Indels	
	Total # comparisons	Concordance rate	Total # comparisons	Concordance rate
Homozygous reference	63,774	0.9995	3,041	0.9884
Heterozygous	65,477	0.9971	3,131	0.9730
Homozygous alternative	64,662	0.9978	3,177	0.9855

Supplementary Table 2c: Genotype concordance for low-frequency variants (MAF 0.005 - 0.05)

WGS compared to WES	SNVs		Indels	
	Total # comparisons	Concordance rate	Total # comparisons	Concordance rate
Homozygous reference	51,007	0.9999	2,610	0.9947
Heterozygous	49,653	0.9985	2,510	0.9648
Homozygous alternative	13,987	0.9954	643	0.9745

Supplementary Table 2d: Genotype concordance for rare variants (MAF < 0.005) but not singleton

WGS compared to WES	SNVs		Indels	
	Total # comparisons	Concordance rate	Total # comparisons	Concordance rate
Homozygous reference	273,922	0.9999	14,605	0.9994
Heterozygous	211,915	0.9992	10,922	0.9848
Homozygous alternative	484	0.9483	22	0.9545

Supplementary Table 2e: Genotype concordance for singletons (Minor Allele Count = 1)

WGS compared to WES	SNVs		Indels	
	Total # comparisons	Concordance rate	Total # comparisons	Concordance rate
Homozygous reference	99,496	0.9999	5,902	0.9999
Heterozygous	70,163	0.9998	4,114	0.9993
Homozygous alternative	0	*None*	0	*None*

Supplementary Table 3: Genotype concordance of DEL and DUP detected from WGS

We compared the concordance of deletions/duplications between WGS-based genotypes with those based on GWA array [13,14] or whole exome sequencing (WES) [11,12] across overlapping variants. Any overlapping variants must have ≥ 50% reciprocal overlap and occur in the same individual. We calculated the overall concordance rate as well as concordance rates when genotypes from the GWAS array or WES are heterozygous and homozygous non-reference.

Comparison	Variant Type	Total overlapping heterozygous & homozygous ALT variants	Number concordant heterozygous & homozygous ALD variants	Concordance
GWAS array	DEL	860	853	0.992
GWAS array	DUP	685	661	0.965
WES	DEL	156	154	0.987
WES	DUP	276	267	0.967

Supplementary Table 4: Summary statistics for repeat expansions detected from WGS

Gene	Gene product	OMIM	Pathological thresholds	Ref for threshold	Range in data	Subjects with pathological repeats
AR	androgen receptor	Spinal and bulbar muscular atrophy	≥40	[15,16]	7-51	3 cases<br>1 control
ATN1	atrophin 1	Dentatorubro-pallidoluysian atrophy	49-88	[15]	8-29	0
ATXN1	ataxin 1	Spinocerebellar ataxia 1	41-83	[15,17]	8-51	4 cases<br>1 control
ATXN10	ataxin 10	Spinocerebellar ataxia 10	400-4,500	[15]	10-22	0
ATXN2	ataxin 2	Spinocerebellar ataxia 2 // Amyotrophic lateral sclerosis	32-200	[15,18]	7-49	2 cases<br>3 controls
ATXN3	ataxin 3	Machado-Joseph disease	52-86	[15]	1-36	0
ATXN7	ataxin 7	Spinocerebellar ataxia 7	>36	[15]	7-22	0
C9orf72	chromosome 9 open reading frame 72	Frontotemporal dementia or amyotrophic lateral sclerosis	Not well established: cases with short (45-80) or long (80-2,000) expansions	[19-21]	1-210	7 cases<br>2 controls
CACNA1A	calcium voltage-gated channel subunit alpha1 A	Epileptic encephalopathy // Episodic ataxia, type 2 // Migraine, familial hemiplegic, 1 // Spinocerebellar ataxia 6	20-33	[15]	4-17	0
CSTB	cystatin B	Epilepsy, progressive myoclonic 1A	30-78	[22,23]	2-14	0
DMPK	dystrophia myotonica protein kinase	Myotonic dystrophy 1	>50	[15]	5-38	0
FMR1	Fragile X mental retardation 1	Fragile X syndrome	200-4,000	[15]	7-54	0
FXN	Frataxin	Fanconi anemia // Friedreich ataxia	70-1,000	[15]	2-64	0
HTT	Huntingtin	Huntington disease	40-180	[24]	7-38	0
JPH3	junctophilin 3	Huntington disease like 2	36-57	[15]	7-19	0
PPP2R2B	protein phosphatase 2 regulatory subunit Bbeta	Spinocerebellar ataxia 12	66-78	[15]	4-29	0

We screened for repeat expansions in 16 genes known to be associated with diseases. Supplementary Table 4 shows the range of repeat units for all subjects and the numbers of subjects that carried potentially pathological repeats.

Supplementary Table 5: Results of burden analysis for ultra-rare SNV/Indels across annotations

Set_Name	Annotation	n_AFF	n_UNAFF	rate_AFF	rate_UNAFF	OR	95% CI	P_emp	FDR_adjust_*P*
coding	LOF	3,678	2,787	3.332	3.026	1.082	(1.034, 1.133)	2.00E-04	0.0049
coding	LOF / exometarget	441	277	0.399	0.301	1.305	(1.124, 1.515)	2.00E-04	0.0049
coding	LOF / exometarget_v6	552	348	0.5	0.378	1.278	(1.12, 1.459)	2.00E-04	0.0049
coding	LOF / LOFintol	602	399	0.545	0.433	1.203	(1.069, 1.355)	5.00E-04	0.0092
coding	LOF / LOFintol_tadatop	30	8	0.027	0.009	2.983	(1.362, 6.53)	0.0011	0.0163
coding	LOF / LOFtol	3,076	2,388	2.786	2.593	1.064	(1.012, 1.119)	0.0068	0.0839
coding	misdmg / exomenontarget	184	122	0.167	0.132	1.272	(1.008, 1.605)	0.0197	0.2083
noncoding	CDTSall_0to1	43,712	35,961	39.594	39.046	1.013	(0.998, 1.027)	0.0381	0.2819
noncoding	CDTSall_0to5_OR_GERP_3to7	345,812	286,419	313.236	310.987	1.005	(1, 1.01)	0.0354	0.2819
noncoding	CDTSall_0to1_OR_GERP_4to7	103,282	85,239	93.553	92.55	1.009	(0.999, 1.018)	0.0342	0.2819
noncoding	constrained / brainexons_CDSrgn	206	144	0.187	0.156	1.2	(0.964, 1.495)	0.0516	0.3373
noncoding	constrained / brainexons_CDSrgn / LOFintol	123	82	0.111	0.089	1.252	(0.945, 1.659)	0.0547	0.3373
coding	synon / LOFtol	7,575	6,147	6.861	6.674	1.025	(0.99, 1.061)	0.0799	0.3517
noncoding	CDTSall_0.5to1	20,561	16,891	18.624	18.34	1.016	(0.995, 1.037)	0.0683	0.3517
noncoding	GERP_3to7	158,940	131,458	143.967	142.734	1.005	(0.998, 1.013)	0.0808	0.3517
noncoding	CDTSall_0.5to1_OR_GERP_4to5	59,907	49,477	54.264	53.721	1.009	(0.997, 1.021)	0.0735	0.3517
noncoding	constrained / TAD_adult	2,177	1,704	1.972	1.85	1.048	(0.983, 1.118)	0.0719	0.3517
coding	synon	10,672	8,719	9.667	9.467	1.019	(0.99, 1.048)	0.0996	0.3685
coding	misnondmg / exometarget	4,804	3,885	4.351	4.218	1.028	(0.985, 1.074)	0.0966	0.3685
noncoding	CDTSall_0to5	199,902	165,712	181.071	179.926	1.004	(0.998, 1.011)	0.0953	0.3685
noncoding	CDTSall_0to0.5_AND_GERP_5to7	250	183	0.226	0.199	1.119	(0.922, 1.359)	0.1171	0.4126
coding	misnondmg	21,520	17,734	19.493	19.255	1.009	(0.99, 1.03)	0.1741	0.4156
coding	misdmg / LOFintol	1,147	911	1.039	0.989	1.043	(0.959, 1.135)	0.1606	0.4156
coding	misdmg / exometarget_v6	1,147	910	1.039	0.988	1.044	(0.96, 1.136)	0.1546	0.4156
noncoding	CDTSall_0.1to0.5	18,892	15,541	17.112	16.874	1.01	(0.989, 1.032)	0.16	0.4156
noncoding	CDTSall_0to0.5	23,151	19,070	20.97	20.706	1.01	(0.991, 1.029)	0.1565	0.4156
noncoding	GERP_3to4	97,487	80,617	88.303	87.532	1.005	(0.996, 1.015)	0.1351	0.4156
noncoding	CDTSall_1to5_OR_GERP_3to4	242,530	201,180	219.683	218.436	1.003	(0.997, 1.009)	0.171	0.4156
noncoding	CDTSall_0.1to0.5_OR_GERP_5to6	37,668	31,038	34.12	33.7	1.008	(0.992, 1.024)	0.1539	0.4156
noncoding	CDTSall_0to0.5_OR_GERP_5to7	43,375	35,762	39.289	38.83	1.008	(0.994, 1.023)	0.1331	0.4156
noncoding	constrained / brainexons	453	349	0.41	0.379	1.07	(0.931, 1.23)	0.1643	0.4156
noncoding	constrained / TSS_2000_upstream / LOFtol	14,987	12,150	13.575	13.192	1.01	(0.988, 1.032)	0.1824	0.4218
noncoding	constrained / HiCloopsBrain_adult / LOFintol	29,564	24,065	26.779	26.129	1.006	(0.992, 1.021)	0.1904	0.427
noncoding	GERP_4to7	61,453	50,841	55.664	55.202	1.005	(0.993, 1.017)	0.1986	0.4322
coding	misnondmg / LOFtol	15,675	12,921	14.198	14.029	1.009	(0.985, 1.032)	0.2306	0.4596
coding	misdmg / LOFintol_tadatop	127	91	0.115	0.099	1.1	(0.847, 1.428)	0.236	0.4596
noncoding	CDTSall_0to5_AND_GERP_3to7	13,030	10,751	11.803	11.673	1.009	(0.983, 1.036)	0.2311	0.4596
noncoding	constrained / adult / loops / endpoints / LOFintol / distal_overlap	11,824	9,576	10.71	10.397	1.01	(0.985, 1.036)	0.2181	0.4596
coding	misnondmg / LOFintol	5,845	4,813	5.294	5.226	1.012	(0.974, 1.052)	0.2735	0.4675

coding	LOF / exomenontarget	161	122	0.146	0.132	1.064	(0.84, 1.347)	0.2969	0.4675
coding	misnondmg / exometarget_v6	5,838	4,812	5.288	5.225	1.011	(0.973, 1.051)	0.2918	0.4675
noncoding	CDTSall_1to5	156,190	129,751	141.476	140.881	1.002	(0.995, 1.009)	0.2899	0.4675
noncoding	GERP_4to5	40,979	33,966	37.119	36.879	1.004	(0.99, 1.019)	0.2726	0.4675
noncoding	GERP_5to6	19,026	15,680	17.234	17.025	1.006	(0.984, 1.028)	0.2866	0.4675
noncoding	GERP_5to7	20,474	16,875	18.545	18.322	1.007	(0.986, 1.028)	0.2642	0.4675
noncoding	constrained / brainexons_CDSrgn / nonLOFintol	83	62	0.075	0.067	1.111	(0.794, 1.554)	0.274	0.4675
noncoding	constrained / adult / loops / endpoints / LOFintol / direct_overlap	17,740	14,489	16.069	15.732	1.006	(0.986, 1.026)	0.2794	0.4675
coding	synon / exometarget	2,545	2,100	2.305	2.28	1.014	(0.955, 1.075)	0.3333	0.5034
noncoding	CDTSall_0to0.1_OR_GERP_6to7	5,707	4,724	5.169	5.129	1.008	(0.97, 1.049)	0.3311	0.5034
noncoding	constrained / HiCloopsBrain_adult	39,360	32,242	35.652	35.008	1.002	(0.99, 1.015)	0.3461	0.5122
noncoding	GERP_6to7	1,448	1,195	1.312	1.298	1.016	(0.938, 1.099)	0.3624	0.5258
coding	misdmg / exometarget	963	789	0.872	0.857	1.012	(0.923, 1.111)	0.3964	0.5535
noncoding	CDTSall_0to0.1	4,259	3,529	3.858	3.832	1.006	(0.962, 1.053)	0.3926	0.5535
coding	synon / LOFintol	3,097	2,572	2.805	2.793	1.005	(0.953, 1.06)	0.4238	0.5808
coding	synon / exometarget_v6	3,093	2,571	2.802	2.792	1.004	(0.952, 1.059)	0.437	0.588
coding	misnondmg / LOFintol_tadatop	397	325	0.36	0.353	1.009	(0.87, 1.17)	0.4543	0.6003
noncoding	constrained / ATACseq_adult	10,953	8,972	9.921	9.742	1	(0.974, 1.027)	0.4886	0.6343
coding	synon / LOFintol_tadatop	238	198	0.216	0.215	0.992	(0.817, 1.204)	0.5363	0.6842
coding	misdmg	3,900	3,259	3.533	3.539	0.995	(0.949, 1.044)	0.5649	0.6956
noncoding	CDTSall_0to1_AND_GERP_4to7	1,883	1,563	1.706	1.697	0.993	(0.927, 1.063)	0.5828	0.6956
noncoding	constrained / FIRE_adult	9,415	7,744	8.528	8.408	0.997	(0.968, 1.027)	0.5753	0.6956
noncoding	constrained / TSS_2000_upstream / LOFintol_tadatop	519	437	0.47	0.474	0.99	(0.873, 1.123)	0.5551	0.6956
noncoding	constrained / loopsAdult / nonLOFintol	15,712	13,090	14.232	14.213	0.997	(0.982, 1.011)	0.67	0.787
coding	synon / exomenontarget	552	472	0.5	0.512	0.969	(0.856, 1.096)	0.6984	0.8075
noncoding	constrained / H3K4me3Brain_adult	66,206	54,715	59.969	59.408	0.997	(0.988, 1.006)	0.7551	0.8597
coding	misdmg / LOFtol	2,753	2,348	2.494	2.549	0.976	(0.924, 1.031)	0.8101	0.8816
noncoding	constrained / brainexons_nonCDSrgn	114	104	0.103	0.113	0.886	(0.676, 1.16)	0.8038	0.8816
noncoding	constrained / H3K27acBrain_adult	71,628	59,142	64.88	64.215	0.997	(0.988, 1.005)	0.7948	0.8816
noncoding	constrained / CTCFBrain_adult	76,058	62,953	68.893	68.353	0.996	(0.988, 1.004)	0.8594	0.9217
coding	misnondmg / exomenontarget	1,041	928	0.943	1.008	0.945	(0.864, 1.033)	0.8958	0.947
noncoding	CDTSall_5to100	4,456,902	3,707,848	4037.049	4025.894	0.994	(0.988, 1)	0.976	0.9907
noncoding	GERP_lt3	4,497,864	3,742,102	4074.152	4063.086	0.993	(0.986, 0.999)	0.9831	0.9907
noncoding	tol	4,310,992	3,587,141	3904.884	3894.833	0.994	(0.989, 0.999)	0.9907	0.9907
noncoding	constrained / TSS_2000_upstream / LOFintol	6,863	5,858	6.216	6.36	0.966	(0.934, 0.999)	0.9812	0.9907

Columns: n_AFF: total number of variants in cases; n_UNAFF: total number of variants in controls; rate_AFF: rate of variants in cases; rate_UNAFF: rate of variants in controls; OR: odds ratio; 95% CI: 95% confidence interval; *P*_emp: One-sided empirical P-values derived by 10,000 permutations by swapping phenotype labels in logistic regression controlling for confounders; FDR_adjusted_*P*: FDR adjusted *P* values obtained by applying the Benjamin and Hochberg FDR method to *P*_emp from the 74 tests listed in Supplementary Table 5 for URVs. Red fonts: significant findings at FDR level of 0.05.

Supplementary Table 6: Genome-wide burden of rare deletions and duplications – Confirmation of prior findings

Higher genome-wide burden of rare CNVs in schizophrenia cases has been repeatedly observed in studies using SNP arrays, which also found that burden was greater for rare CNVs that were deletions, larger, or rarer [14,25,26]. We verified this general pattern of prior findings using WGS SV calls. The burden of the largest DEL and DUP (> 500 kb) was increased to the greatest extent in cases versus controls, consistent with prior studies, but was not significant due to small numbers of events.

MAF	SV type	n_ AFF	n_ UNAFF	Rate AFF	Rate UNAFF	OR	95% CI	*P*_emp
Rare (<0.01)	DEL	18,232	14,254	15.69	15.23	1.03	(1 , 1.05)	0.0074
Rare (<0.01)	DUP	4,101	3,289	3.529	3.514	1.00	(0.96 , 1.05)	0.4292
Rare (<0.01)	DEL & > 500kb	15	8	0.01291	0.0085	1.509	(0.64 , 3.56)	0.1909
Rare (<0.01)	DUP & > 500kb	43	29	0.03701	0.03098	1.202	(0.746 , 1.94)	0.2232
Ultra-rare	DEL	3,923	2,886	3.376	3.083	1.086	(1.04 , 1.14)	0.0001
Ultra-rare	DUP	1,090	827	0.938	0.8835	1.06	(0.972 , 1.16)	0.0920

Columns: n_AFF: total number of variants in schizophrenia cases; n_UNAFF: total number of variants in controls; rate_AFF: rate of variants in schizophrenia cases; rate_UNAFF: rate of variants in controls; OR: odds ratio; 95% CI: 95% confidence interval; *P*_emp: One-sided empirical *P*-values derived by 10,000 permutations by swapping phenotype labels in logistic regression controlling for confounders.

Supplementary Table 7: Results of burden analysis of ultra-rare SVs: genome-wide burden

SV Type	CDS Type	n_ AFF	n_UNAFF	Rate AFF	Rate UNAFF	OR	95% CI	P_emp	FDR_adjusted_*P*
DEL	.all	3,923	2,886	3.376	3.083	1.086	(1.04 , 1.14)	0.0001	0.0029
DUP	.all	1,090	827	0.938	0.884	1.06	(0.972 , 1.16)	0.0920	0.2052
INV	.all	430	299	0.370	0.319	1.015	(0.967 , 1.07)	0.2903	0.4009
DEL	.coding	515	357	0.443	0.382	1.165	(1.02 , 1.33)	0.0132	0.0555
DUP	.coding	316	238	0.272	0.254	1.069	(0.907 , 1.26)	0.2183	0.3332
INV	.coding	48	29	0.041	0.031	1.327	(0.845 , 2.08)	0.1181	0.2308
DEL	.noncoding	3,408	2,529	2.933	2.702	1.079	(1.03 , 1.13)	0.0011	0.0160
DUP	.noncoding	774	589	0.666	0.629	1.060	(0.954 , 1.18)	0.1393	0.2376
INV	.noncoding	382	270	0.329	0.289	1.012	(0.965 , 1.06)	0.3256	0.4292

Columns: CDS type: '.all"=both coding and noncoding SVs; '.coding"=coding SVs only, ".noncoding"=noncoding SVs only; n_AFF: total number of variants in schizophrenia cases; n_UNAFF: total number of variants in controls; rate_AFF: rate of variants in schizophrenia cases; rate_UNAFF: rate of variants in controls; OR: odds ratio; 95% CI: 95% confidence interval; *P*_emp: One-sided empirical P-values derived by 10,000 permutations by swapping phenotype labels in logistic regression controlling for confounders; FDR_adjusted_*P*: FDR adjusted *P* values obtained by applying the Benjamin and Hochberg FDR method to *P*_emp from the total of 29 tests for ultra-rare SVs listed in Supplementary Tables 7 and 8. Red fonts: significant findings at FDR level of 0.05.

Supplementary Table 8: Results of burden analysis for ultra-rare SVs: across annotations

Annotations	n_AFF	n_UNAFF	rate_AFF	rate_UNAFF	OR	95% CI	*P*_emp	FDR_adjusted_*P*
TADboundaries.AdultBrain	95	44	0.082	0.047	1.613	(1.13 , 2.3)	0.0037	0.0283
TADboundaries.FetalBrain	93	45	0.080	0.048	1.581	(1.11 , 2.25)	0.0039	0.0283
CTCF	5,321	3,899	4.579	4.166	1.325	(1.05 , 1.67)	0.0092	0.0534
H3K27ac	5,034	3,678	4.332	3.929	1.125	(1.01 , 1.25)	0.0149	0.0555
H3K4me3	5,169	3,783	4.448	4.042	1.176	(1.02 , 1.35)	0.0153	0.0555
CELF4_HiC.loops.int	871	598	0.750	0.639	1.103	(0.989 , 1.23)	0.0356	0.1147
CHD8_HiC.loops.int	799	555	0.688	0.593	1.089	(0.973 , 1.22)	0.0668	0.1937
HiC.loops.AdultBrain	1,538	1,102	1.324	1.177	1.064	(0.974 , 1.16)	0.0824	0.2052
LoFintolerent_HiC.loops.int	994	702	0.855	0.750	1.073	(0.968 , 1.19)	0.0881	0.2052
RBFOX_HiC.loops.int	747	527	0.643	0.563	1.073	(0.954 , 1.21)	0.1194	0.2308
PSD_HiC.loops.int	577	407	0.497	0.435	1.076	(0.942 , 1.23)	0.1365	0.2376
genes.ID/DD/ASD_HiC.loops.int	171	116	0.147	0.124	1.102	(0.876 , 1.39)	0.2019	0.3253
Ca+Channel_HiC.loops.int	19	11	0.016	0.012	1.302	(0.631 , 2.69)	0.2504	0.3631
FMRP_HiC.loops.int	433	318	0.373	0.340	1.029	(0.887 , 1.19)	0.3459	0.4361
PSD95_HiC.loops.int	77	56	0.066	0.060	1.063	(0.755 , 1.5)	0.3638	0.4396
FIRE.AdultBrain	218	160	0.188	0.171	1.032	(0.841 , 1.27)	0.3819	0.4430
SCZGWAS_HiC.loops.int	82	65	0.071	0.069	0.966	(0.706 , 1.32)	0.5863	0.6540
CMCqval05_HiC.loops.int	232	183	0.200	0.196	0.951	(0.785 , 1.15)	0.6912	0.7378
NMDARC_HiC.loops.int	54	45	0.046	0.048	0.894	(0.604 , 1.32)	0.7124	0.7378
ATACseq.AdultBrain	1,297	1,001	1.116	1.069	0.971	(0.885 , 1.06)	0.7398	0.7398

Note:

1. Variants included in the analysis are ultra-rare DEL, DUP, or INV that overlapped ≥ 10% of the given functional elements.
2. Columns: n_AFF: total number of variants in schizophrenia cases; n_UNAFF: total number of variants in controls; rate_AFF: rate of variants in schizophrenia cases; rate_UNAFF: rate of variants in controls; OR: odds ratio; 95% CI: 95% confidence interval; *P*_emp: One-sided empirical *P*-values derived by 10,000 permutations by swapping phenotype labels in logistic regression controlling for confounders; FDR_adjusted_*P*: FDR adjusted *P* values obtained by applying the Benjamin and Hochberg FDR method to *P*_emp from the total of 29 tests for ultra-rare SVs listed in Supplementary Tables 7 and 8. Red fonts: significant findings at FDR level of 0.05.

Supplementary Table 9: Burden of ultra-rare TADs-affecting SVs – by overlapping criterion

SV Type	CDS Type	Extent TAD boundaries overlapped by SVs	Name in Fig S14	n_ AFF	n_ UNAFF	Rate AFF	Rate UNAFF	OR	95% CI	P_emp
DELDUPINV	.all	Any (≥1bp)	TADbou.adult_any	158	104	0.136	0.111	1.135	(0.885 , 1.46)	0.1525
DELDUPINV	.all	Any (≥1bp)	TADbou.fetal_any	163	110	0.140	0.118	1.128	(0.887 , 1.43)	0.1616
DELDUPINV	.all	>10%	TADbou.adult_0.1	95	44	0.082	0.047	1.613	(1.13 , 2.3)	0.0037
DELDUPINV	.all	>10%	TADbou.fetal_0.1	93	45	0.080	0.048	1.581	(1.11 , 2.25)	0.0039
DELDUPINV	.all	>30%	TADbou.adult_0.3	58	22	0.050	0.024	2.014	(1.23 , 3.29)	0.0013
DELDUPINV	.all	>30%	TADbou.fetal_0.3	56	27	0.048	0.029	1.621	(1.02 , 2.57)	0.0191
DELDUPINV	.coding	Any (≥1bp)	TADbou.adult_any	55	33	0.047	0.035	1.269	(0.828 , 1.94)	0.1349
DELDUPINV	.coding	Any (≥1bp)	TADbou.fetal_any	52	31	0.045	0.033	1.283	(0.828 , 1.99)	0.1369
DELDUPINV	.coding	>10%	TADbou.adult_0.1	44	21	0.038	0.022	1.580	(0.946 , 2.64)	0.0370
DELDUPINV	.coding	>10%	TADbou.fetal_0.1	42	20	0.036	0.021	1.605	(0.95 , 2.71)	0.0340
DELDUPINV	.coding	>30%	TADbou.adult_0.3	31	15	0.027	0.016	1.595	(0.857 , 2.97)	0.0697
DELDUPINV	.coding	>30%	TADbou.fetal_0.3	25	15	0.022	0.016	1.302	(0.684 , 2.48)	0.2280
DELDUPINV	.noncoding	Any (≥1bp)	TADbou.adult_any	103	71	0.089	0.076	1.072	(0.785 , 1.46)	0.3259
DELDUPINV	.noncoding	Any (≥1bp)	TADbou.fetal_any	111	79	0.096	0.084	1.065	(0.798 , 1.42)	0.3325
DELDUPINV	.noncoding	>10%	TADbou.adult_0.1	51	23	0.044	0.025	1.649	(1.01 , 2.69)	0.0181
DELDUPINV	.noncoding	>10%	TADbou.fetal_0.1	51	25	0.044	0.027	1.571	(0.971 , 2.54)	0.0347
DELDUPINV	.noncoding	>30%	TADbou.adult_0.3	27	7	0.023	0.007	3.015	(1.31 , 6.94)	0.0030
DELDUPINV	.noncoding	>30%	TADbou.fetal_0.3	31	12	0.027	0.013	2.024	(1.04 , 3.96)	0.0167

Supplementary Table 10 Burden of ultra-rare TADs-affecting SVs– by variant type

SV Type	CDS Type	Overlap Criterion	Name in Fig S15	n_ AFF	n_ UNAFF	Rate AFF	Rate UNAFF	OR	95% CI	P_emp
DEL	.all	>10%	TADbou.adult_0.1	43	18	0.037	0.019	1.773	(1.02 , 3.08)	0.0178
DEL	.all	>10%	TADbou.fetal_0.1	44	18	0.038	0.019	1.848	(1.07 , 3.2)	0.0117
DUP	.all	>10%	TADbou.adult_0.1	38	21	0.033	0.022	1.383	(0.808 , 2.37)	0.1183
DUP	.all	>10%	TADbou.fetal_0.1	34	20	0.029	0.021	1.321	(0.754 , 2.32)	0.1654
INV	.all	>10%	TADbou.adult_0.1	14	5	0.012	0.005	2.235	(0.805 , 6.21)	0.0668
INV	.all	>10%	TADbou.fetal_0.1	15	7	0.013	0.007	1.713	(0.697 , 4.21)	0.1326

Supplementary Table 11: Size distribution of ultra-rare TADs-affecting SVs in schizophrenia cases

Type	Total number of variants	Found from GWA array?	Number (%)	Minimum	1st Quartile	Median	Mean	3rd Quartile	Maximum
DEL	43	Yes	12 (27.9%)	32,459 bp	120,414 bp	180,976 bp	285,746 bp	339,836 bp	856,078 bp
		No	31 (72.1%)	4,015 bp	5,239 bp	7,660 bp	33,722 bp	10,406 bp	783,290 bp
DUP	38	Yes	20 (52.6%)	59,038 bp	181,519 bp	372,107 bp	403,930 bp	556,488 bp	977,667 bp
		No	18 (47.4%)	5,116 bp	8,819 bp	16,540 bp	28,162 bp	22,028 bp	125,847 bp

Supplementary Table 12 Genomic features nearby ultra-rare TADs-affecting SVs in schizophrenia cases

Type	Total number of variants	Size Range (kb)	Number of variants overlapped any genes	Number of variants overlapped genes with high pLI scores	Number of variants overlapped SCZ risk genes	Number of variants overlapped ID/DD/ASD risk genes	Number of variants overlapped genes with high pLI scores or implicated in SCZ/ID/DD/ASD	Number of variants connected to genes with high pLI or genes implicated in SCZ/ID/DD/ASD via a HCRCI
DEL	43	4 – 856	30	4	0	4	7 (16.3%)	8 (18.6%)
DUP	38	5 – 977	25	12	1	8	17 (44.7%)	14 (36.8%)
INV	14	4.5–995	12	8	1	3	8 (57.1%)	7 (50.0%)

Abbreviations: pLI: the probability that a gene is intolerant to a Loss of Function (LoF) mutation; SCZ: schizophrenia; ID: intellectual disability; DD: developmental delay; ASD: autism; HCRCI: a high-confidence regulatory chromatin interaction.

Supplementary Figures

Supplementary Figure 1: Distribution of mean depth of coverage

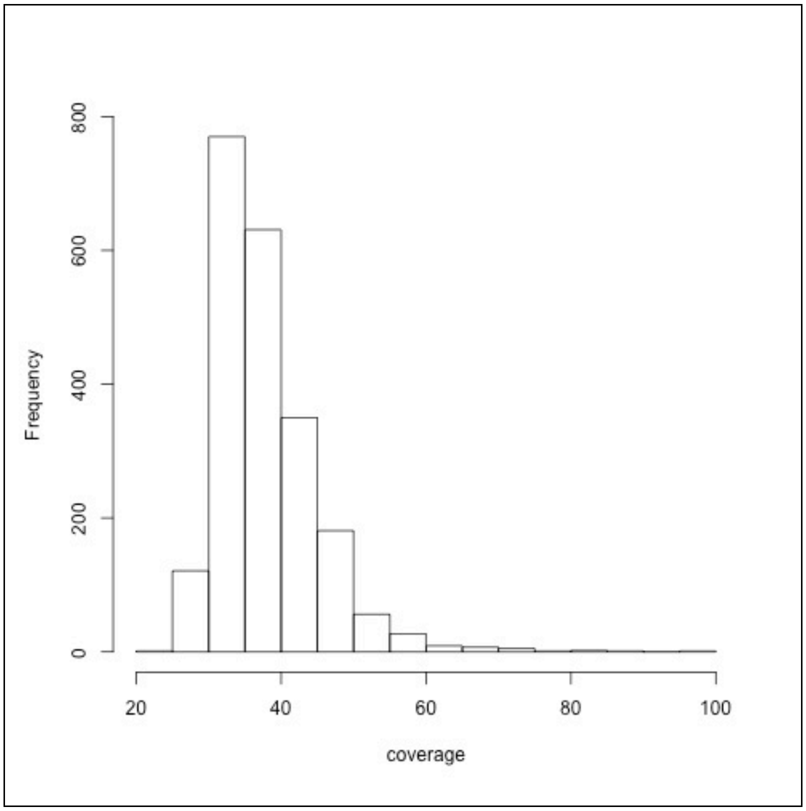

Distribution of mean depth of coverage for all 2,098 samples.

Supplementary Figure 2: Percentage of bases covered versus depth of coverage

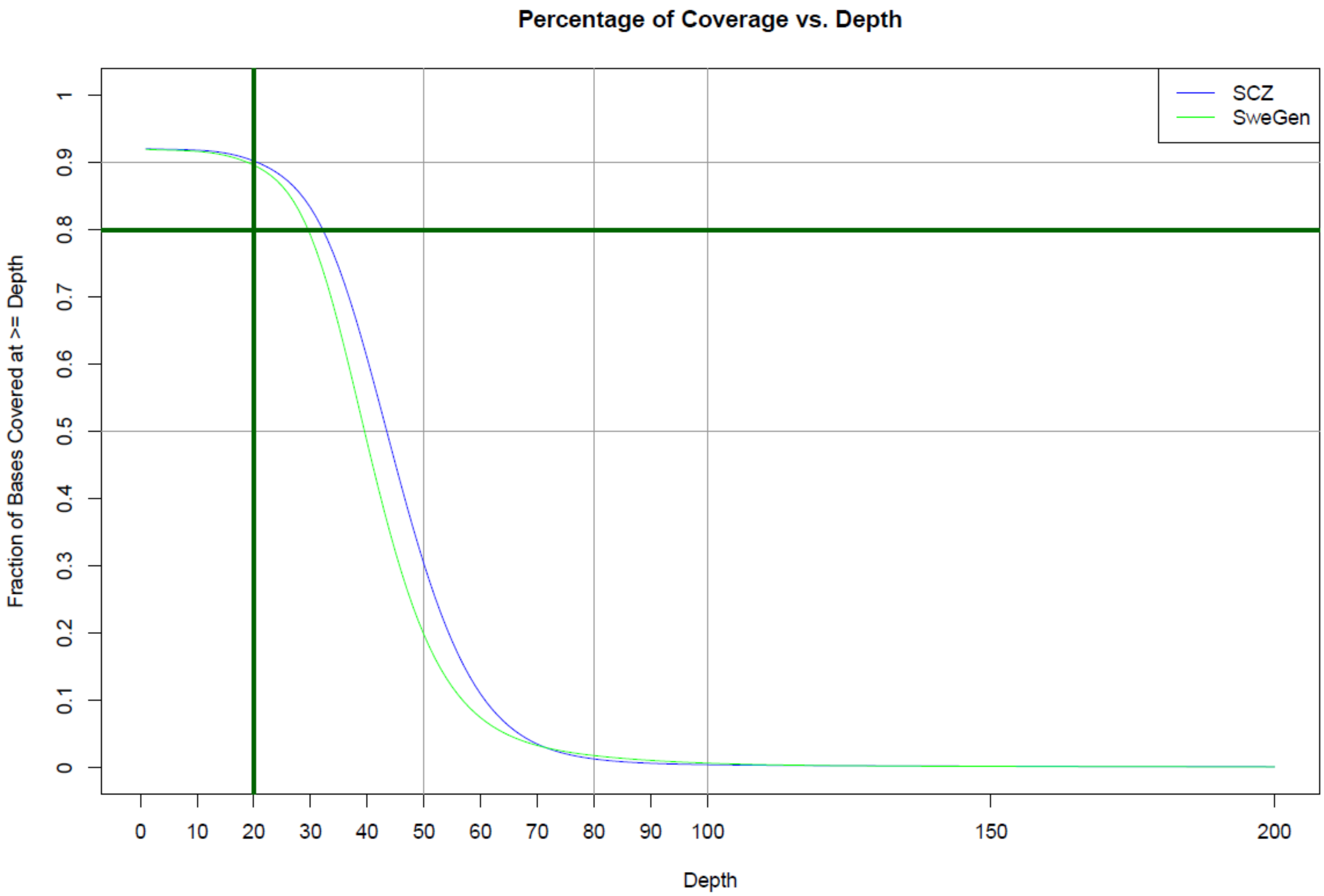

Mean fraction of bases covered deeper than a certain depth threshold as a function of depth of coverage for cases and controls. As a cutoff for samples with good coverage, we selected at ≥ 80% of bases should be covered ≥ 20 times for a confident variant calling.

Supplementary Figure 3: Ancestry principal component plots

We combined biallelic autosomal SNPs from the 2,098 subjects of our case/control sample with 1000 Genomes Project [27] and performed PCA after SNP quality control.

Abbreviation: ACB=African-Caribbean (N=102); ASW=African-American SW (N=104); LWK=Luhya (N=115); YRI=Yoruba (N=185). CLM=Colombian (N=104); MXL=Mexican-American (N=103); PEL=Peruvian (N=104); PUR=Puerto Rican (N=105). CDX=Dai Chinese (N=100); CHB=Han Chinese (N=108); CHS=Southern Han Chinese (N=153); JPT=Japanese (N=105); KHV=Kinh Vietnamese (N=121). South Asian: GIH=Gujarati (N=113). CEU=CEPH (N=180); FIN=Finnish (N=99); GBR=British (N=103); IBS=Spanish (N=149); TSI=Tuscan (N=109). NSPHS=Northern Swedish Population Health Study (N=58).

Supplementary Figure 3a. WGS samples in global context

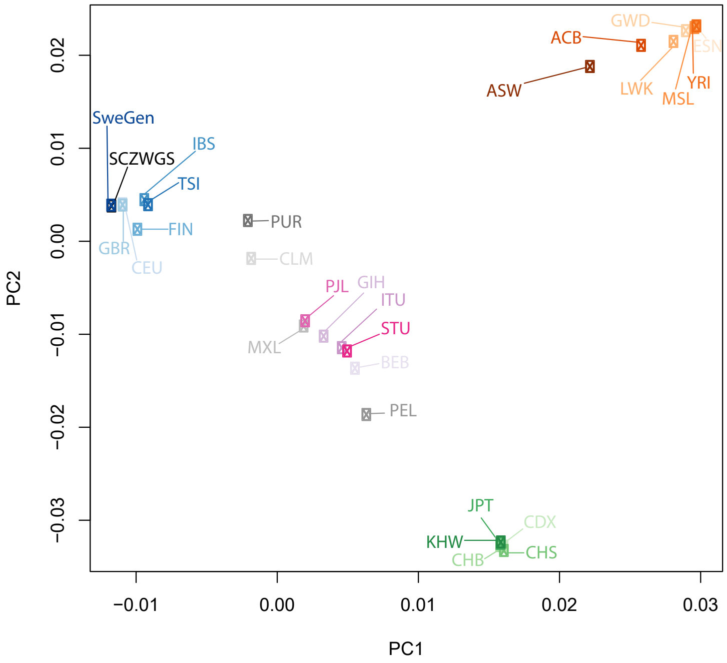

Ancestry PCs for Swedish SCZ cases and controls with 1000 Genomes Project cohorts for comparison. Group medians are shown. Swedish cases (SCZWGS) and controls (SweGen) cluster tightly with other European samples.

Supplementary Figure 3b. WGS samples in European context

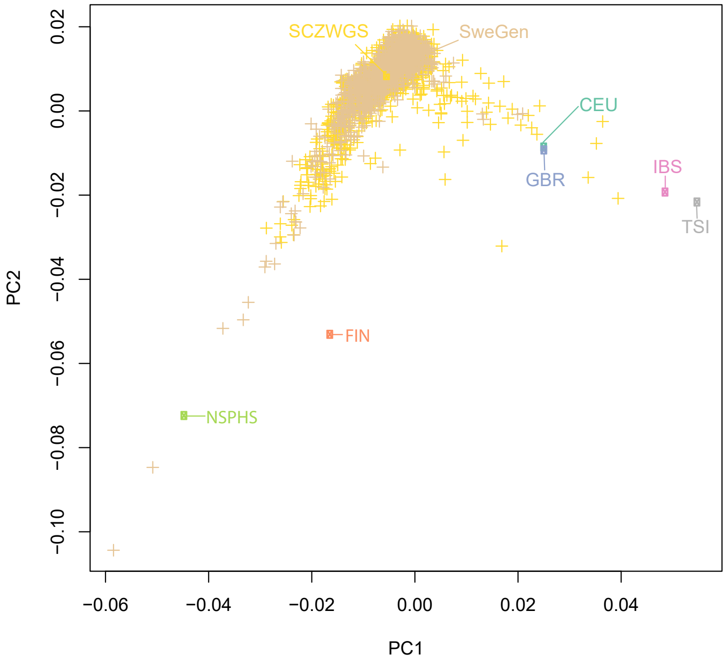

Ancestry PCs for SCZ case/control cohort, NSPHS, and European samples from 1000 Genomes. Group medians are shown for NSPHS and 1000 Genomes samples. We combined the biallelic autosomal SNPs from Swedish cases and controls, NSPHS, and European samples from 1000 Genomes, and then performed a new PCA.

Supplementary Figure 3c. WGS samples in Swedish context

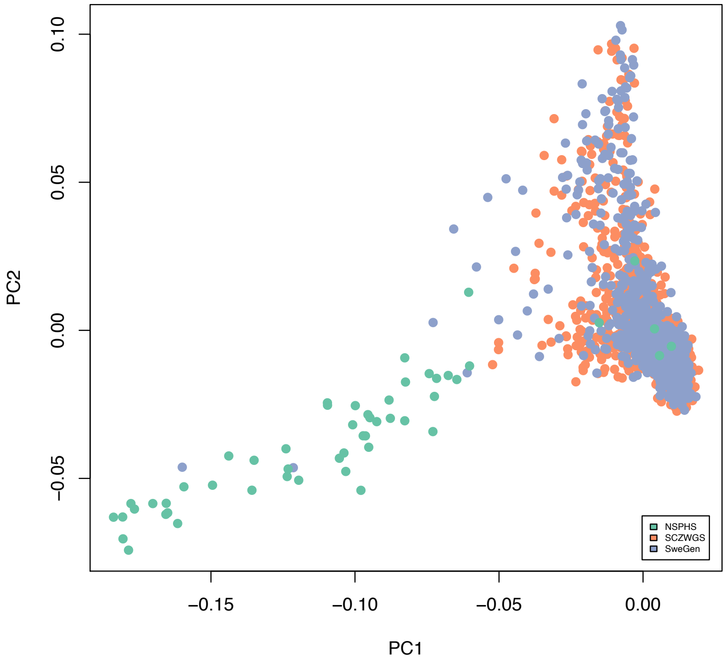

Ancestsry PCs for SCZ case/control cohort and NSPHS. We combined the biallelic autosomal SNPs, and then performed a new PCA.

Supplementary Figure 4: Size distribution of DEL, DUP, INV

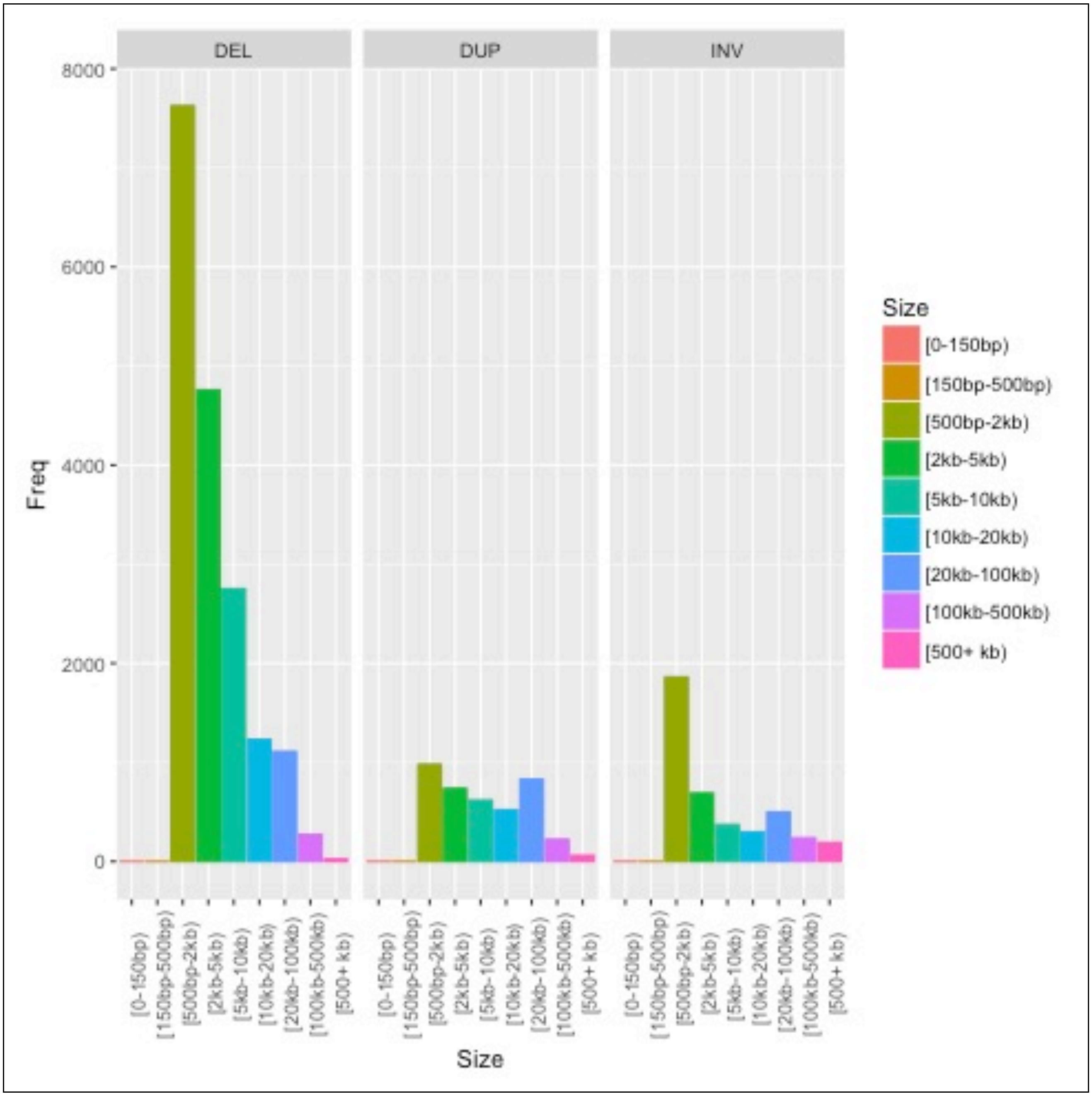

The sizes of DEL, DUP and INV ranged from 500bp to 1Mbp, with a median size of 2,592bp for DEL, a median size of 7,179bp for DUP, and a median size of 3,265bp for INV.

Supplementary Figure 5: Size distribution of MEI

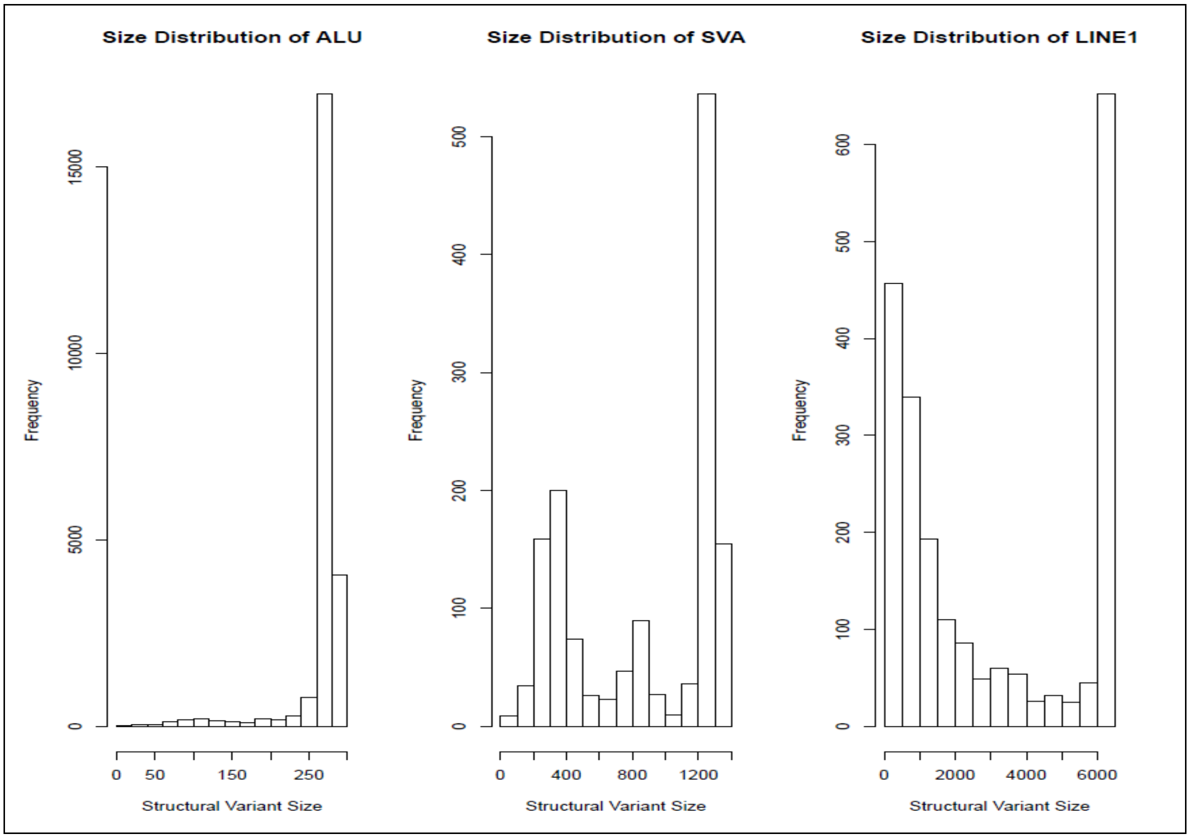

For MEI, the size range for ALU was 15bp to 281bp (median 279bp), for SVA was 38-1,316bp (median 1,162bp), and for LINE1 was 23bp to 6,019bp (median 1,780bp).

Supplementary Figure 6: Power calculation for association analysis

Power calculation for association analyses given our sample size (1,162 cases and 936 controls) was conducted using the R/gap package (v1.2.1). We assumed an additive model, lifetime risk of schizophrenia of 1%, type I error level of $5x10^{-8}$ (Supplementary Figure 6a), or $1x10^{-5}$ (Supplementary Figure 6b). We computed the minimal detectable genotypic relative risk to achieve 20%, 80% power over a range of frequency of risk alleles in the population. For single-variant association test, the X-axis of the power plot represents the frequency of a single variant. For burden test, the X-axis of the power plots represents the aggregated frequency of a set of variants aggregated for a target region.

Supplementary Figure 6a Assuming type I error level of $5x10^{-8}$

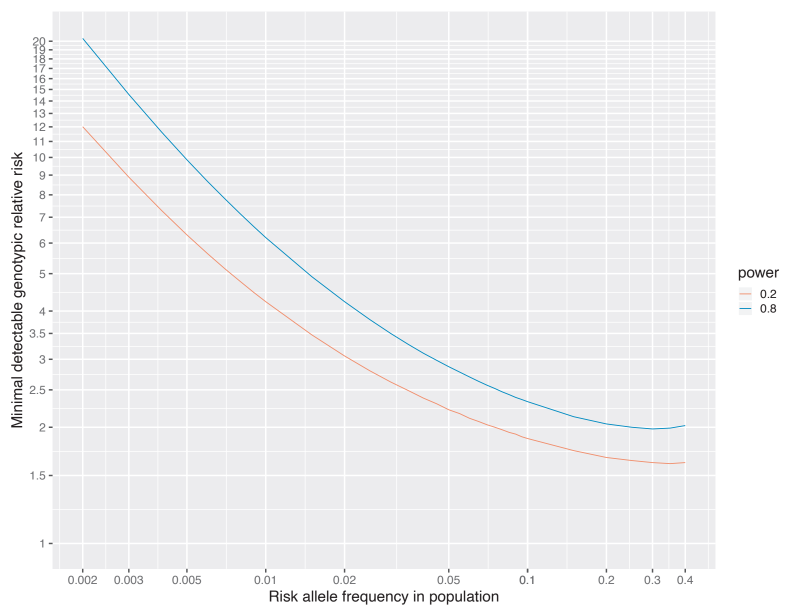

Supplementary Figure 6b Assuming type I error level of $1x10^{-5}$

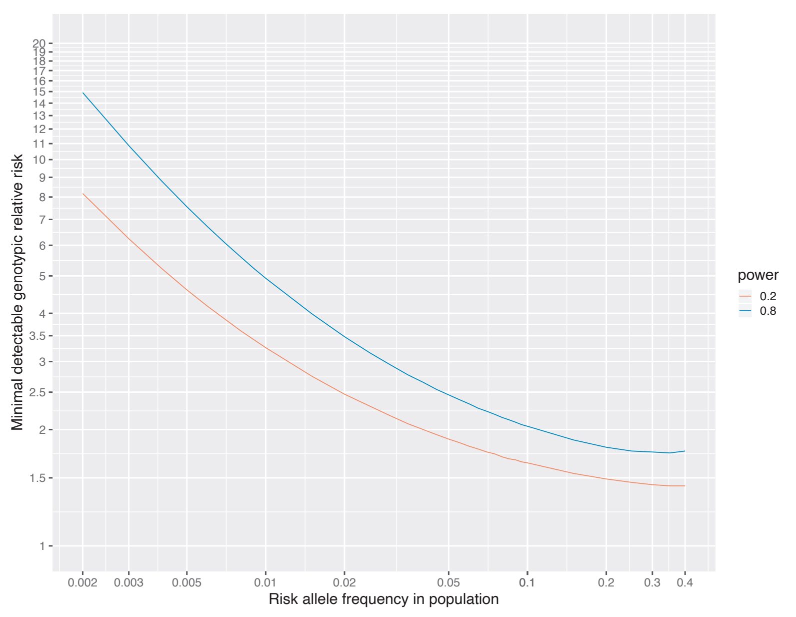

Supplementary Figure 7: Density plots of ultra-rare SNV/indel counts before and after outlier pruning

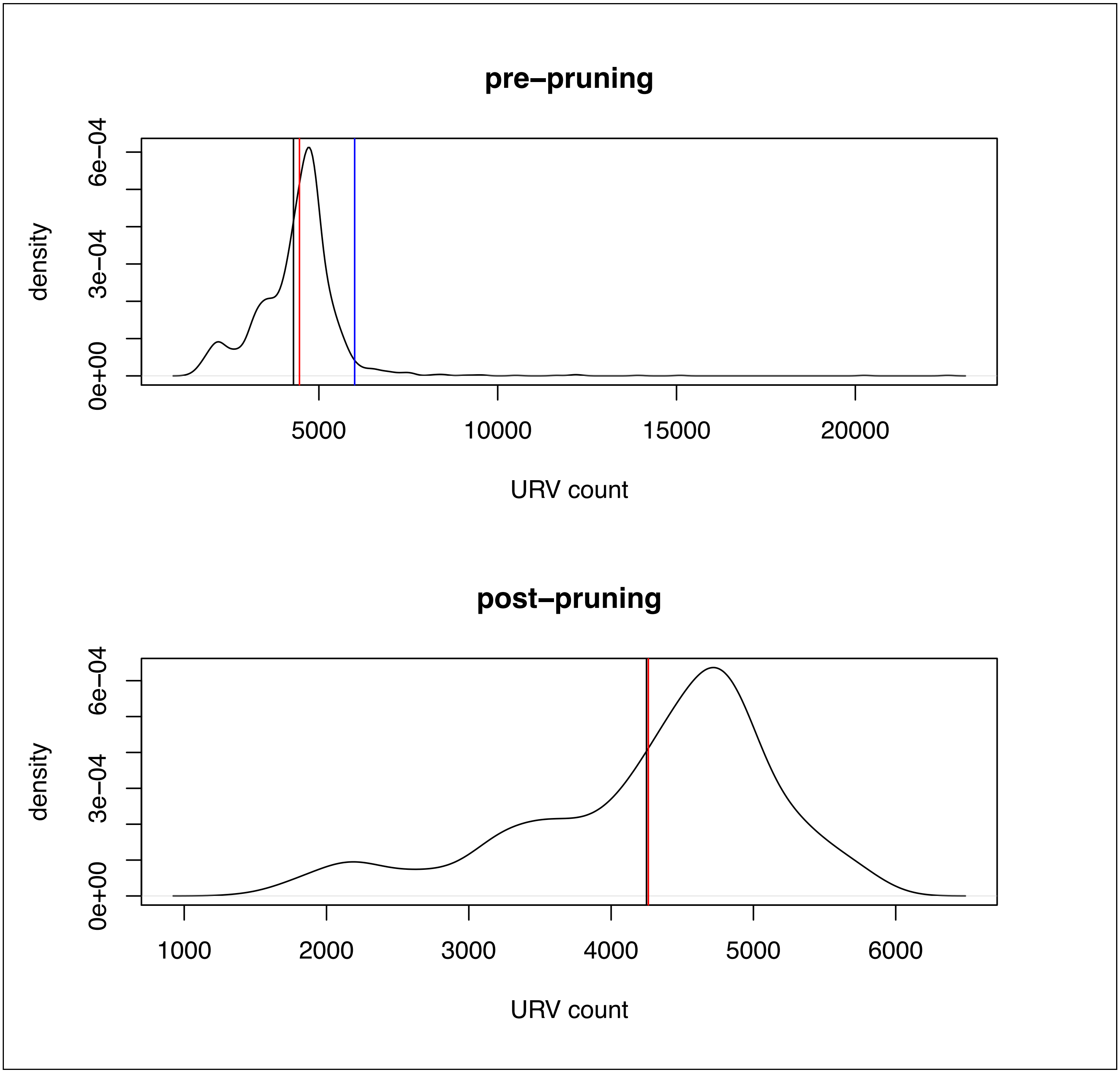

The figure shows the distribution of URV counts across SNVs and indels before (top) and after (bottom) outlier pruning. Before outlier pruning (top panel), with 1,162 cases and 936 controls, we fitted a simple logistic regression model with case/control status as the dependent variable and count per sample of URVs as the predictor variable. We found that cases had a higher mean URV count (4,456 vs. 4,289, $P = 0.002$, two-sided uncorrected), and that this was primarily driven by the presence of a portion of samples with unusually high URV counts. The red vertical line indicates mean URV count in case and the black vertical line indicates mean URV count in controls. The blue vertical line indicates the threshold (>6,000) used to determine URV outlier status. After outlier pruning (bottom panel), we had 1,104 cases and 921 controls and there was no evidence for a difference in mean URV count between cases and controls after this pruning step was carried out (4,262 vs 4,249, $P = 0.4225$, one-sided assuming cases have higher URV count, uncorrected).

Supplementary Figure 8: URV outlier status and principal components

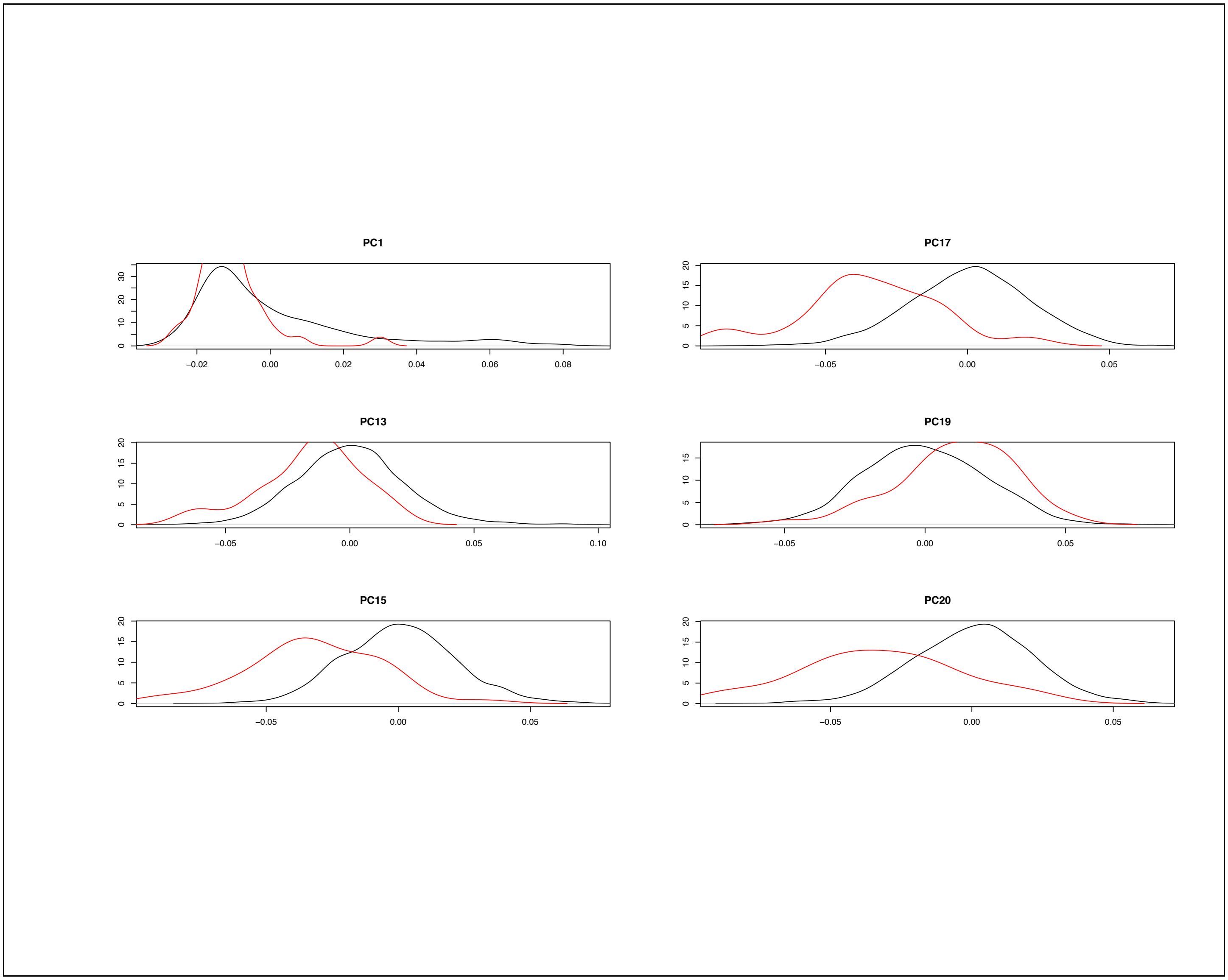

Density plots for URV count outliers (red) vs non-outliers (black) where several common variant PC axes cleanly segregated outliers vs non-outliers. The outlier samples may have an unusually high URV count because of the presence of variation which is uncommon in the main Swedish cohort due to relatively higher ancestry heterogeneity, similar to the previous finding from the full Swedish sample in Genovese et al [12].

Supplementary Figure 9: Burden of coding URV in LoF-intolerant genes overlapping old and new Agilent kit loci

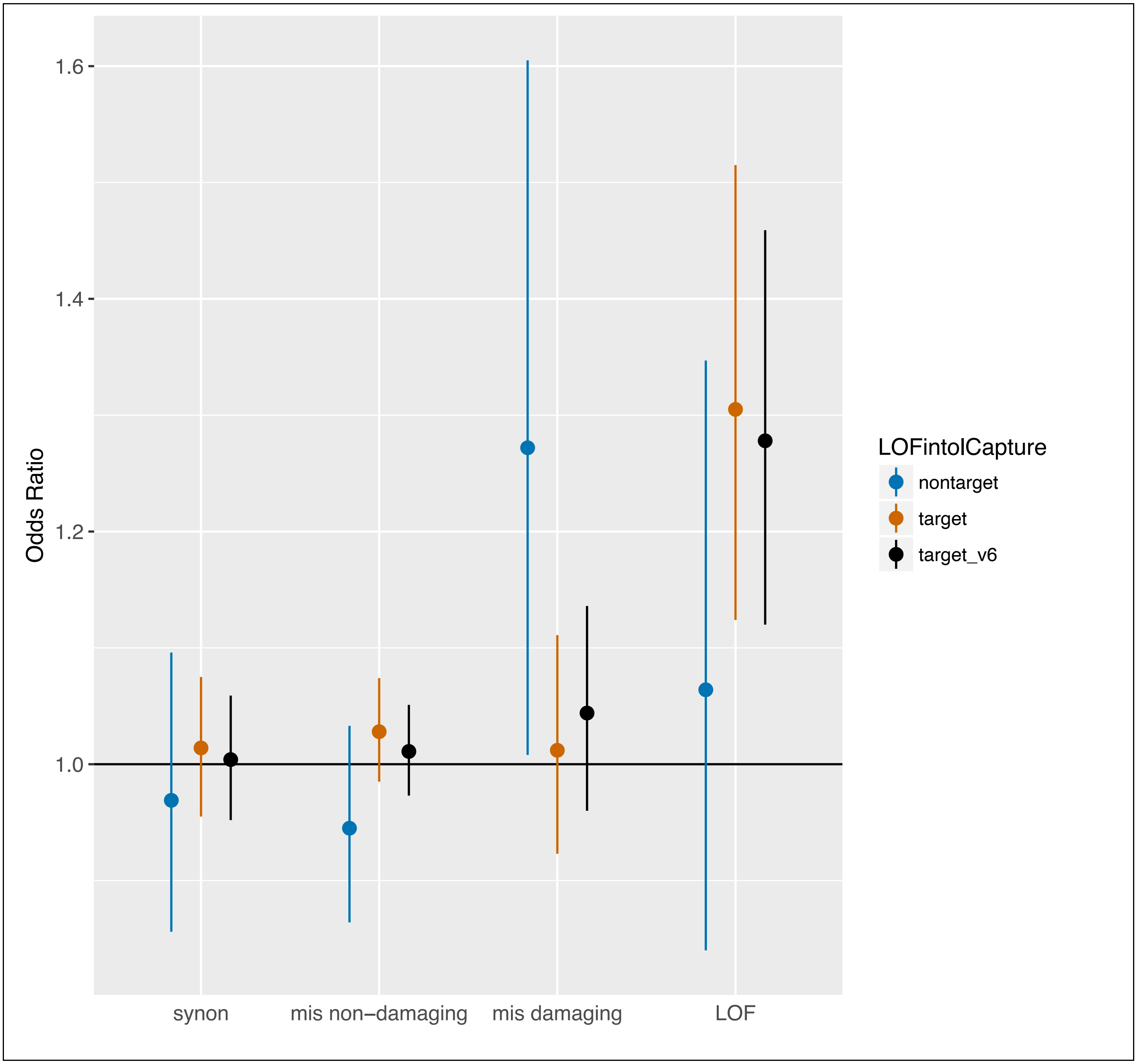

The figure depicts the burden of coding URVs that affect LoF intolerant genes and are within old Agilent kit loci as part of previously published exome Agilent kit case/control data ('target') and newer Agilent kit loci ('target_v6'). The Y-axis indicates odds ratio. For each specific burden test, we use a vertical line to indicate the 95% confidence interval of odds ratio and a dot at the center of the line to indicate the point estimate of odds ratio.

Supplementary Figure 10: Burden of noncoding URVs across binned regions by sequence constraint

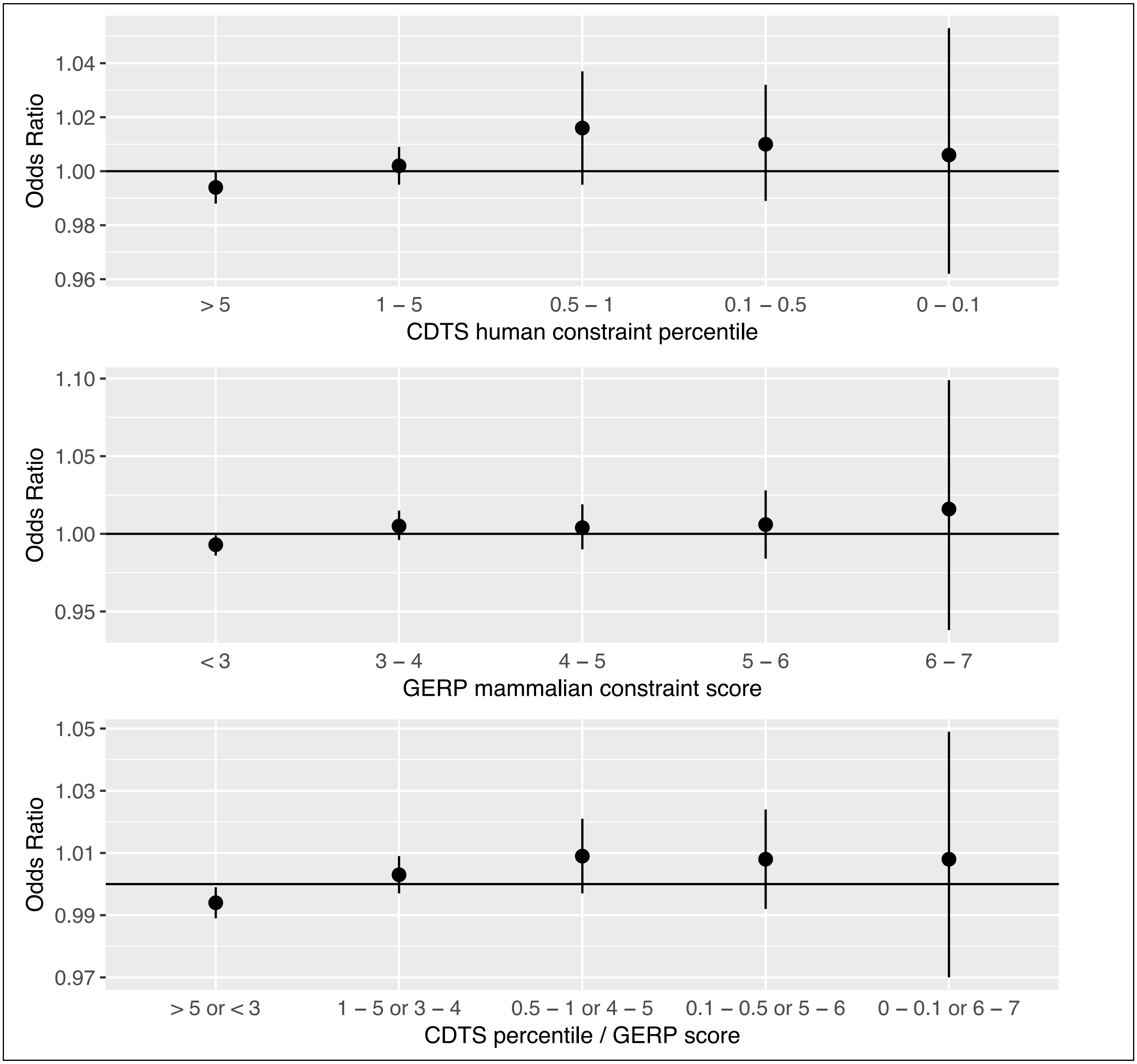

The figure shows the case/control URV burden across different bins of constraint across humans (CDTS percentile, top) and mammals (GERP score, middle). For each specific burden test, we use a vertical line to indicate the 95% confidence interval of odds ratio and a dot at the center of the line to indicate the point estimate of odds ratio. We do not observe any individual bin where there is a level of case excess that survives multiple testing correction. On the bottom, we define union bins based on the union between CDTS and GERP bins. For subsequent tests on functional annotations we subset on variants that are in regions with CDTS < 1%, or GERP >= 4.

Supplementary Figure 11: Burden of noncoding URVs in constrained nucleotides in brain functional annotations

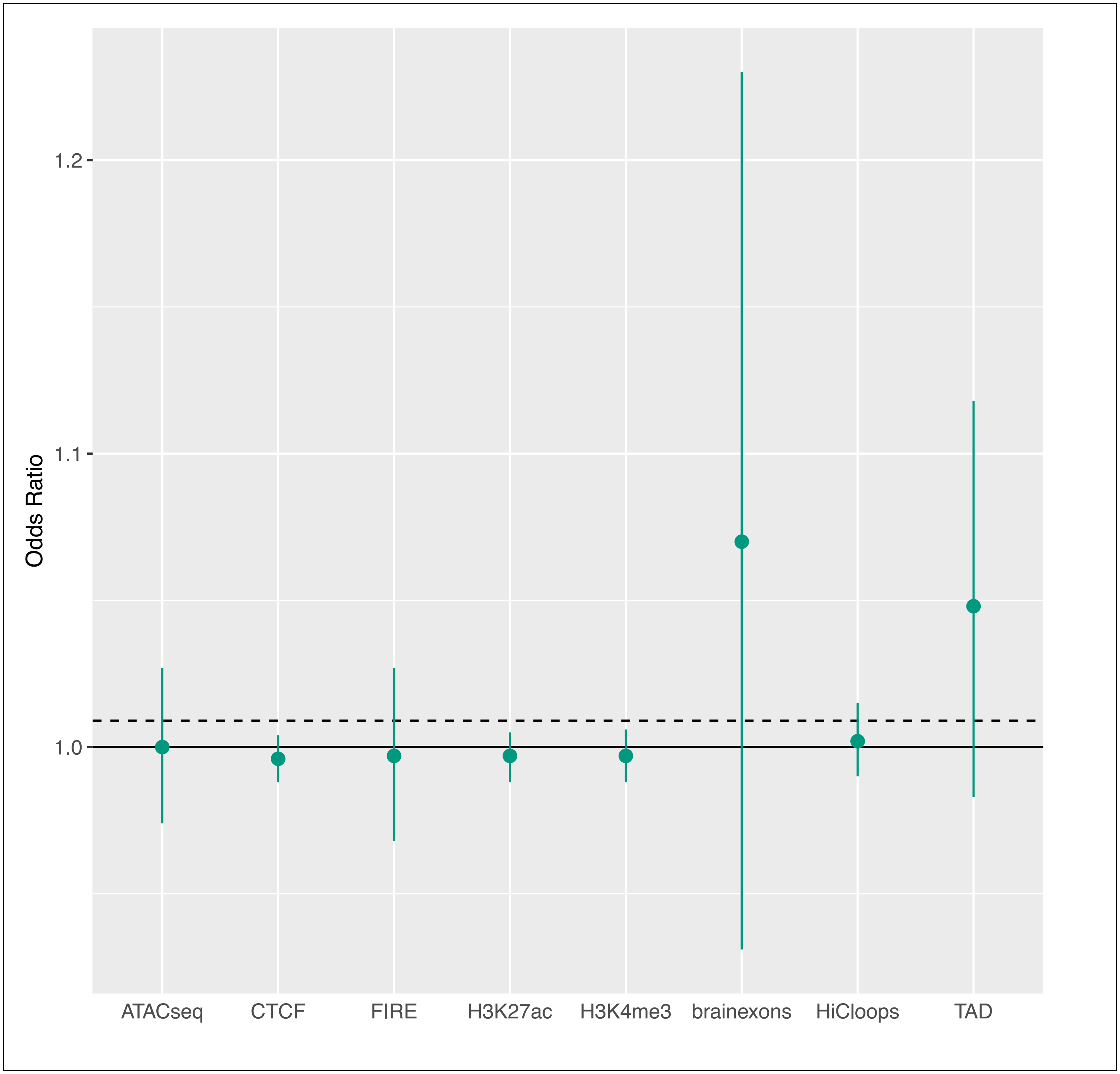

The figure shows the results of case/control URV burden tests within constrained regions (CDTS < 1%, or GERP >= 4) that overlap listed functional loci, all of which are derived from adult brain. The Y-axis indicates odds ratio. For each specific burden test, we use a vertical line to indicate the 95% confidence interval of odds ratio and a dot at the center of the line to indicate the point estimate of odds ratio. The dashed horizontal line represents the baseline enrichment for URVs with GERP >= 4 or CDTS < 1%.

Supplementary Figure 12: Genome-wide burden of ultra-rare SVs

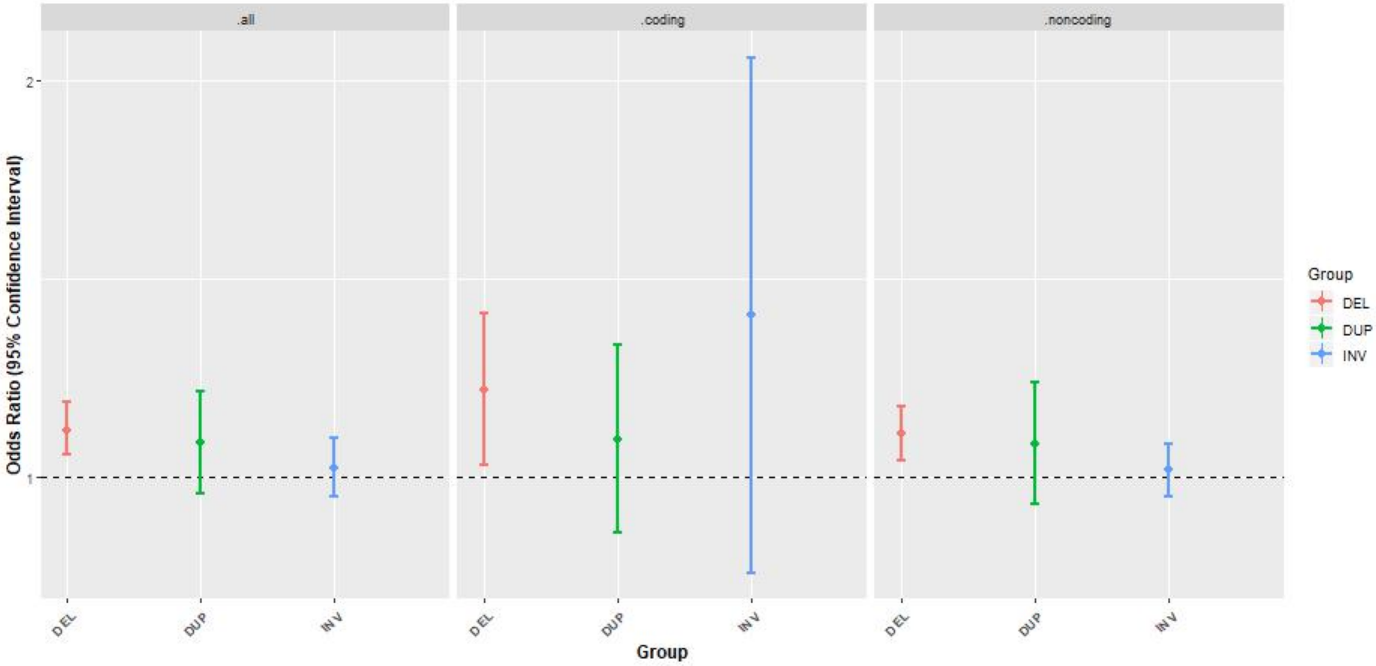

The three panels from the left to the right indicate burden tests done for ".all" - any SVs (coding or noncoding); ".coding" - coding SVs; ".noncoding" - noncoding SVs. Within each panel, the Y-axis indicates odds ratio, and the X-axis indicates variant type: DEL, DUP, INV. For each specific burden test, we use a vertical line to indicate the 95% confidence interval of odds ratio and a dot at the center of the line to indicate the point estimate of odds ratio.

Supplementary Figure 13: Distribution of coding versus noncoding ultra-rare SVs

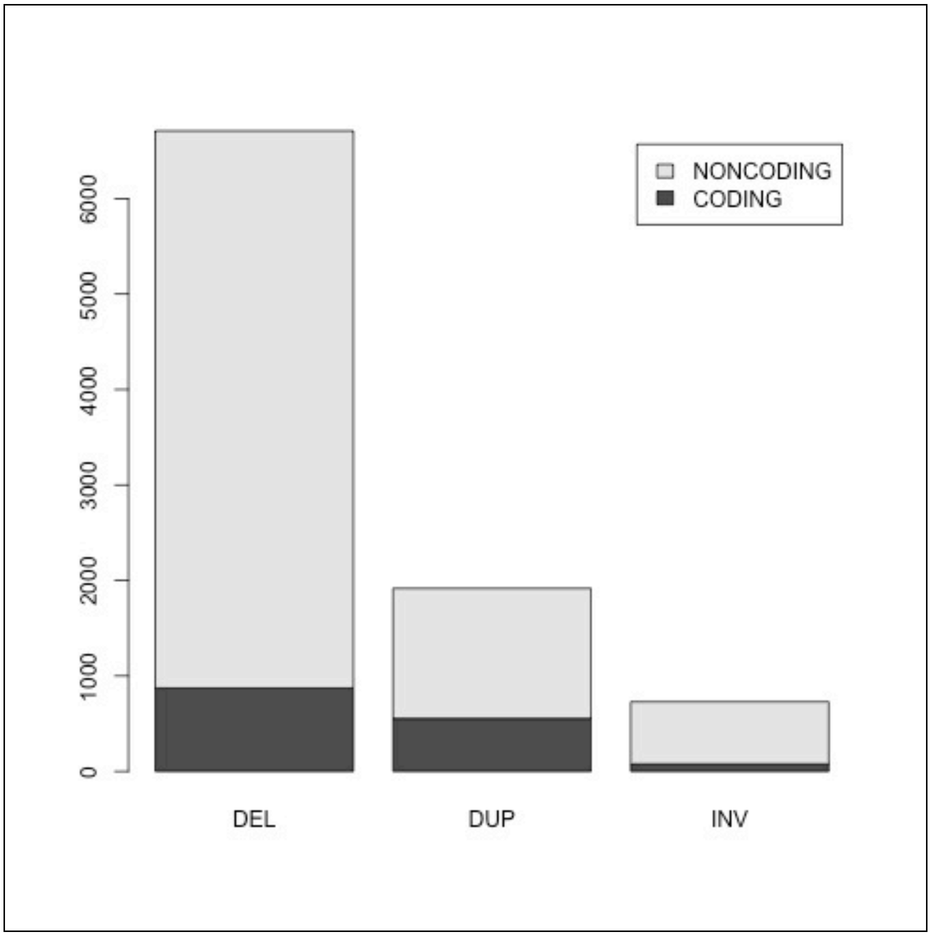

In this barplot, the X-axis indicates variant type: DEL, DUP, INV; and the Y-axis indicates the number of ultra-rare variants of a given type, coding or noncoding. Of these ultra-rare SVs, the vast majority were noncoding: DEL - 872 coding, 5,937 noncoding (87%); DUP - 554 coding, 1,363 noncoding (71%); INV - 77 coding, 652 noncoding (89%).

Supplementary Figure 14: Burden of ultra-rare TADs-affecting SVs – by overlap criterion

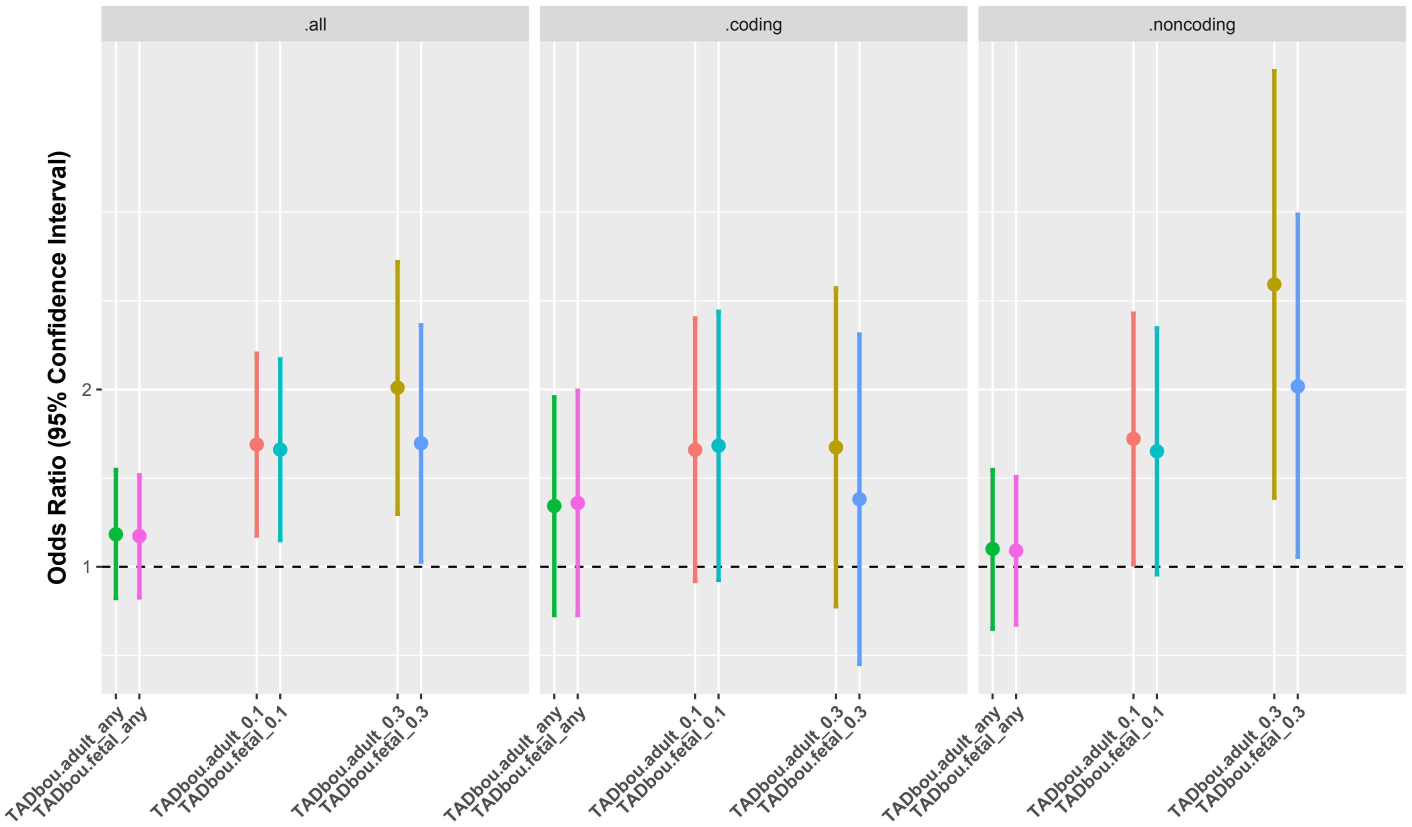

The three panels from the left to right indicates burden tests done for ".all" - any SVs (coding or noncoding); ".coding" - coding SVs; ".noncoding" - noncoding SVs.

Within each panel, the Y-axis indicates odds ratio. For each specific burden test, we use a vertical line to indicate the 95% confidence interval of odds ratio and a dot at the center of the line to indicate the point estimate of odds ratio.

Legends below the figure indicate specific class of variants that the burden tests were performed: "TADbou.adult_any": SVs had any (≥1bp) overlap with TAD boundaries in adult brain; "TADbou.fetal_any": SVs had any (≥1bp) overlap with TAD boundaries in fetal brain; "TADbou.adult_0.1": SVs overlapped > 10% of TAD boundaries in adult brain; "TADbou.fetal_0.1": SVs overlapped > 10% of TAD boundaries in fetal brain; "TADbou.adult_0.3": SVs overlapped > 30% of TAD boundaries in adult brain; "TADbou.fetal_0.3": SVs overlapped > 30% of TAD boundaries in fetal brain.

Supplementary Figure 15: Burden of ultra-rare TADs-affecting SVs – by variant type

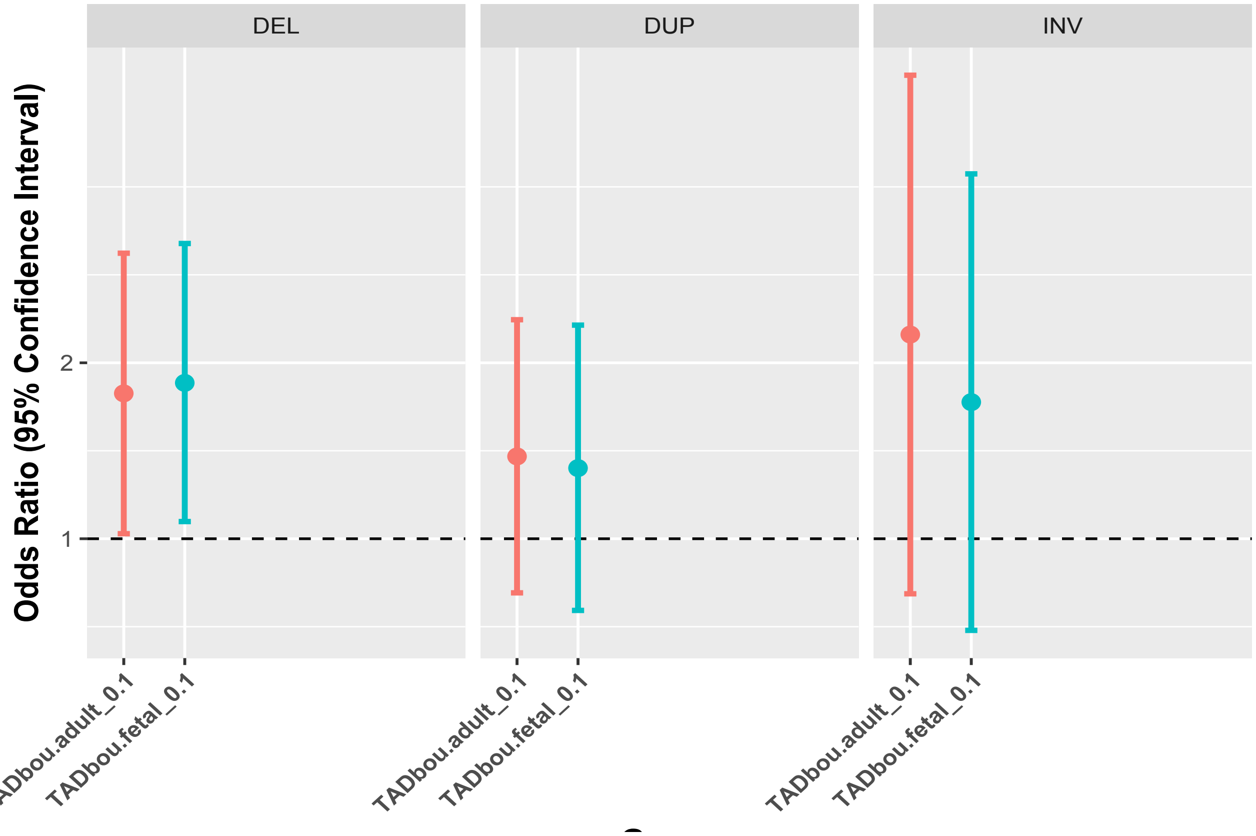

The three panels from the left to right indicates burden tests done for DEL, DUP, INV.

Within each panel, the Y-axis indicates odds ratio. For each specific burden test, we use a vertical line to indicate the 95% confidence interval of odds ratio and a dot at the center of the line to indicate the point estimate of odds ratio.

Legends below the figure indicate specific class of variants that the burden tests were performed: "TADbou.adult_0.1": SVs overlapped > 10% of TAD boundaries in adult brain; "TADbou.fetal_0.1": SVs overlapped > 10% of TAD boundaries in fetal brain;

Supplementary Figure 16: IGV plot for a deletion that overlapped TAD boundaries and was confirmed by GWA SNP array †

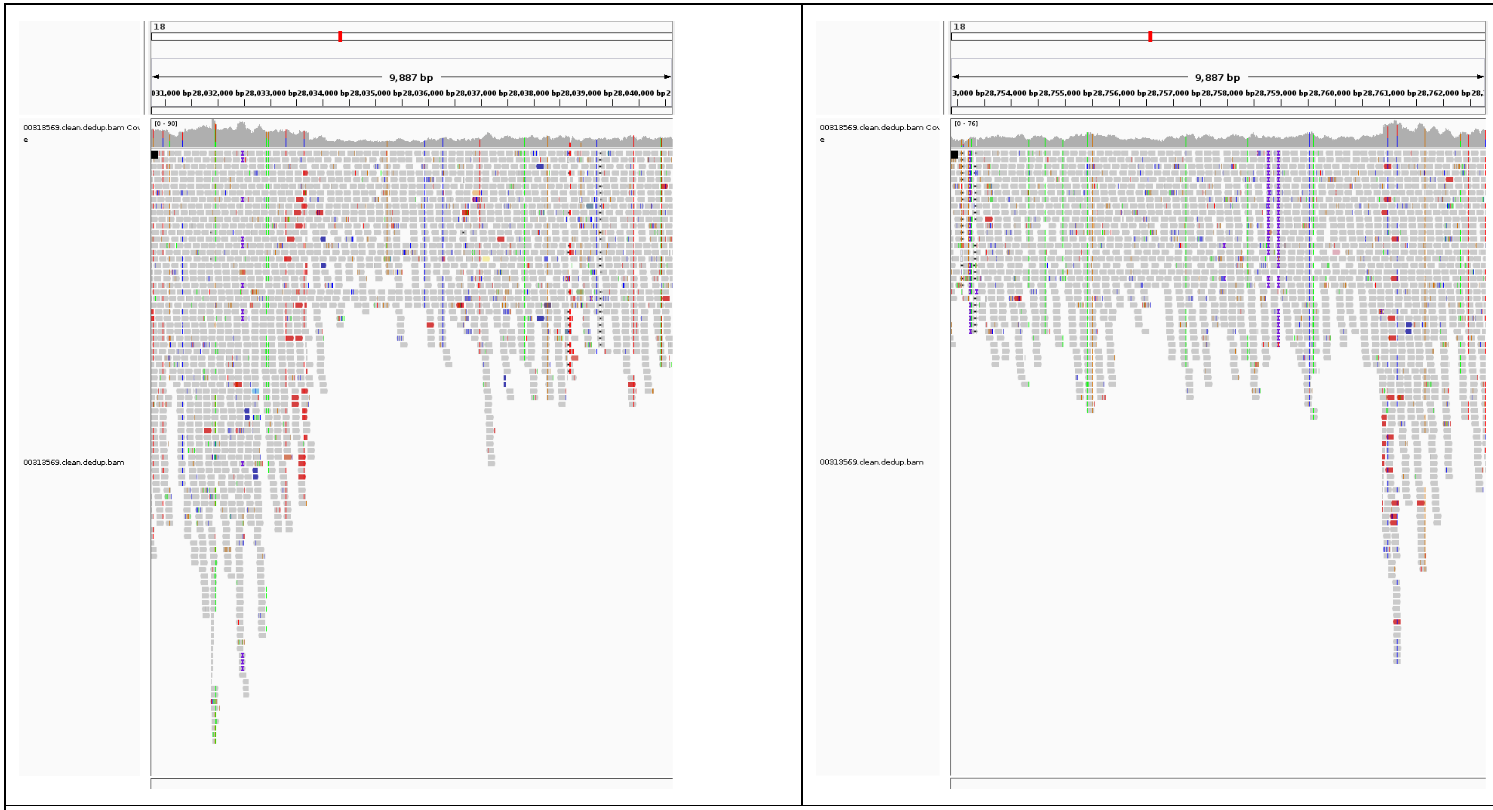

IGV plot for start and end of a deletion that overlapped TAD boundaries and was confirmed by SNP array (chr18:28,033,736-28,760,884).

† *Indications of a deletion: a sudden drop in coverage, noticeable cliff at the start and end positions with some homozygous alternates within the deletion. Red reads are also indicative of a deletion. Indications of a duplication: a sudden increase in coverage, noticeable cliff at the start and end positions with some heterozygous alternates, approximately (1/3 and 2/3 in proportion) within the duplication. Green reads represent a tandem duplication.*

Supplementary Figure 17: IGV plot for a duplication that overlapped TAD boundaries and was confirmed by GWA SNP array

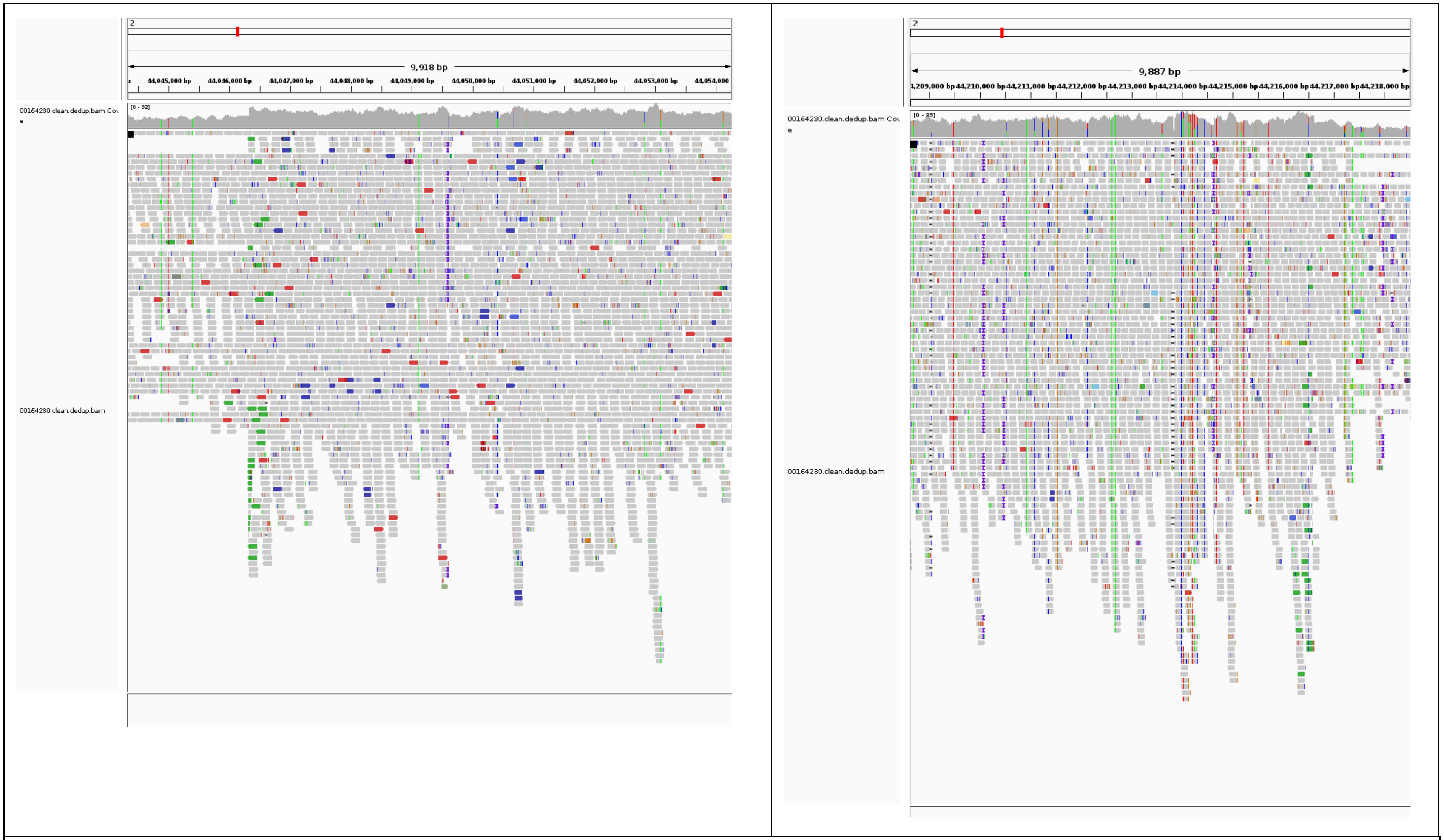

IGV plot for start and end of a duplication that overlapped TAD boundaries and is confirmed with GWAS (chr2: 44,046,326-44,216,616)

Supplementary Figure 18: IGV plot for a deletion that overlapped TAD boundaries and was not found by GWA SNP array

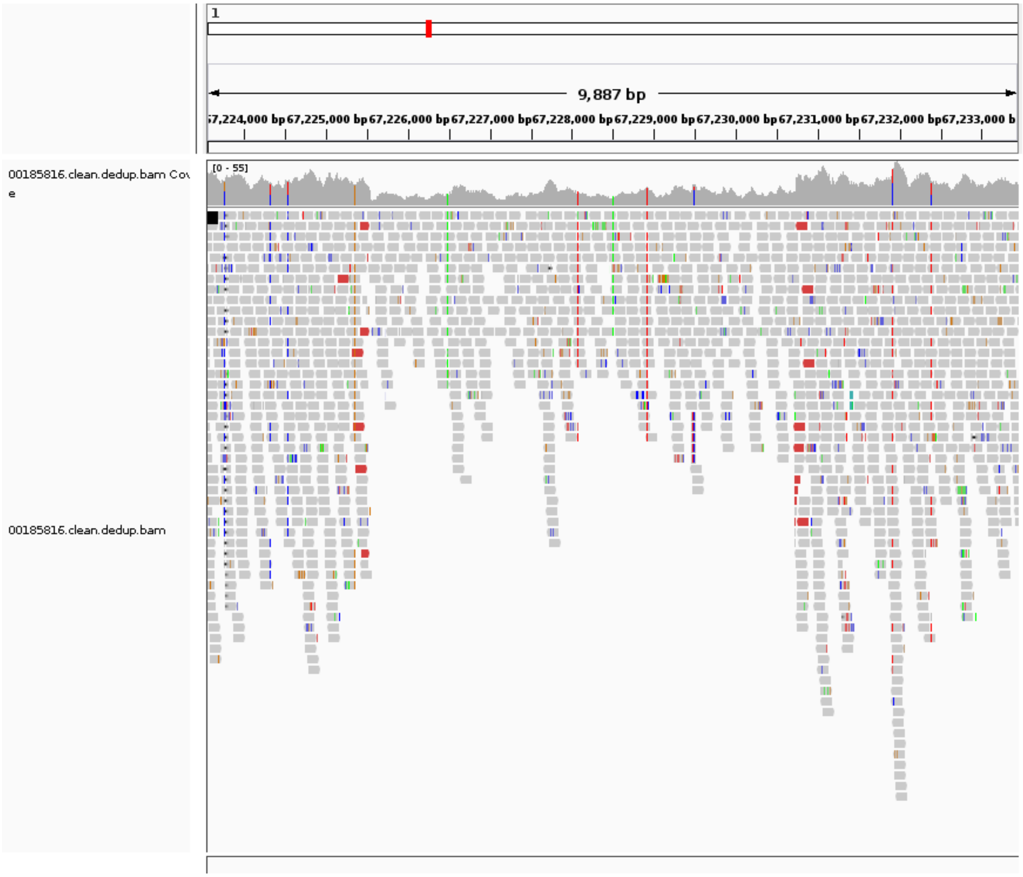

IGV plot of a deletion that overlapped TAD boundaries and was not found from GWAS array. chr1:67,225,545-67,230,729

Supplementary Figure 19: IGV plot for a duplication that overlapped TAD boundaries and was not found by GWA SNP array

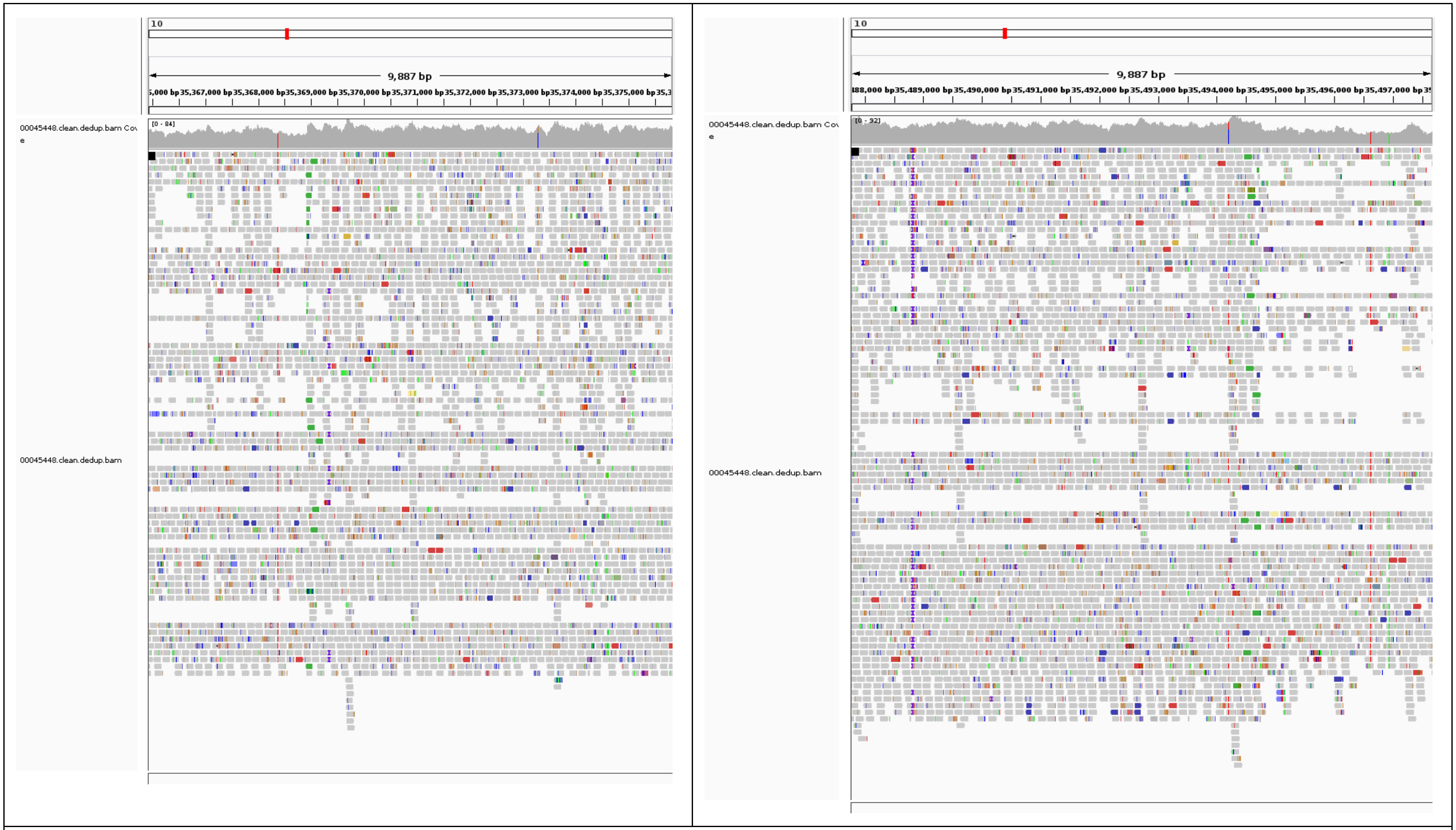

IGV plot for start and end of a duplication that overlapped TAD boundaries and was not found from GWAS array (chr10:35,368,922-35,494,769)

Supplementary Figure 20: QQ and Manhattan plots for common SNVs and indels

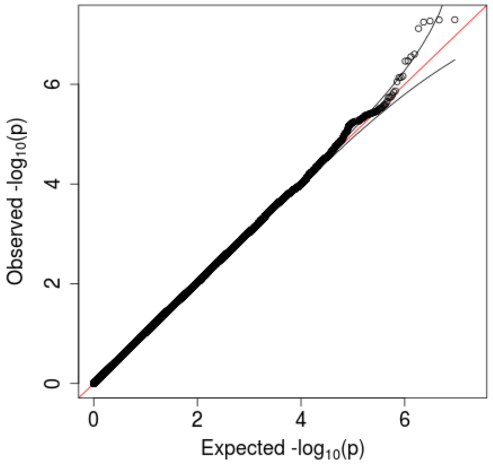

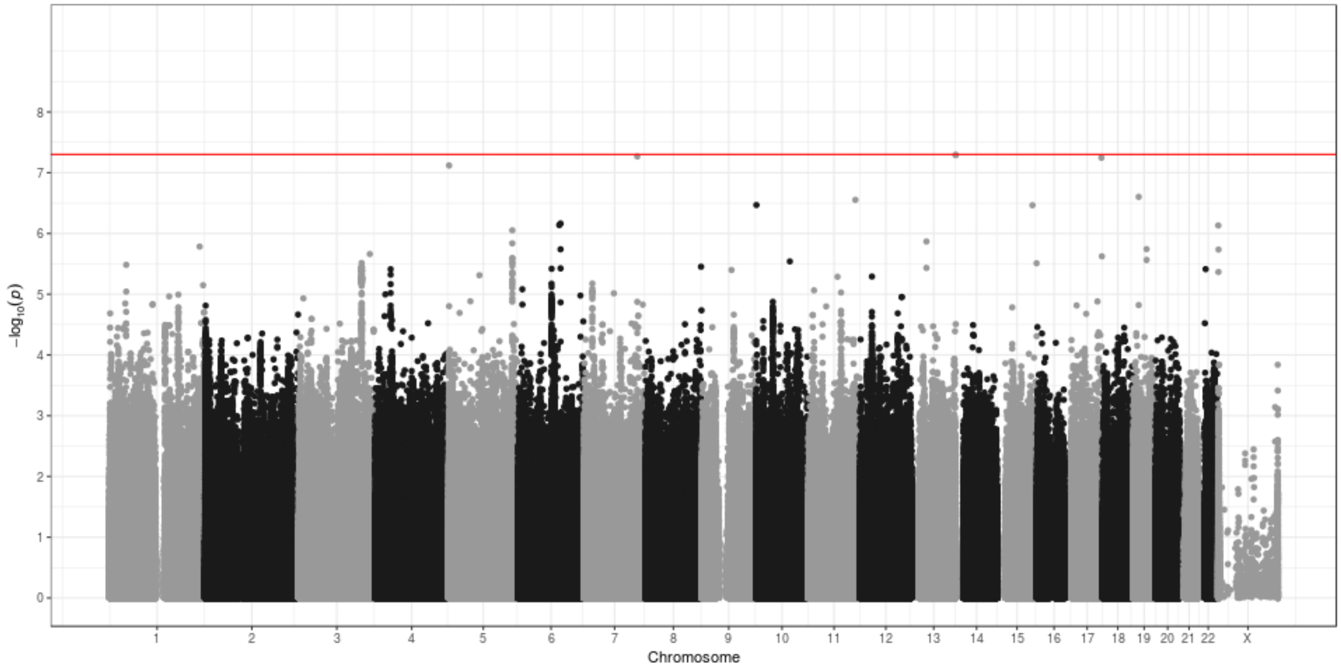

QQ and Manhattan plots for chr1-22, X for all SNVs and indels (9,263,808 variants). We obtain a λ_{GC} of 1.03. A logistic regression model with additive genetic model (Plink --logistic) with empirically determined covariates (PC2 as covariate for chr1-22, PC2 and sex as covariates for chrX) was used to estimate association between single variants and schizophrenia. Statistical tests are two-sided. *P* values shown in the figures are the asymptotic *P* values from Plink outputs. To correct for multiple comparisons in the analysis of common variant association, we used the established genome-wide significance threshold of $5x10^{-8}$.

Supplementary Figure 21: QQ and Manhattan plots for common DEL

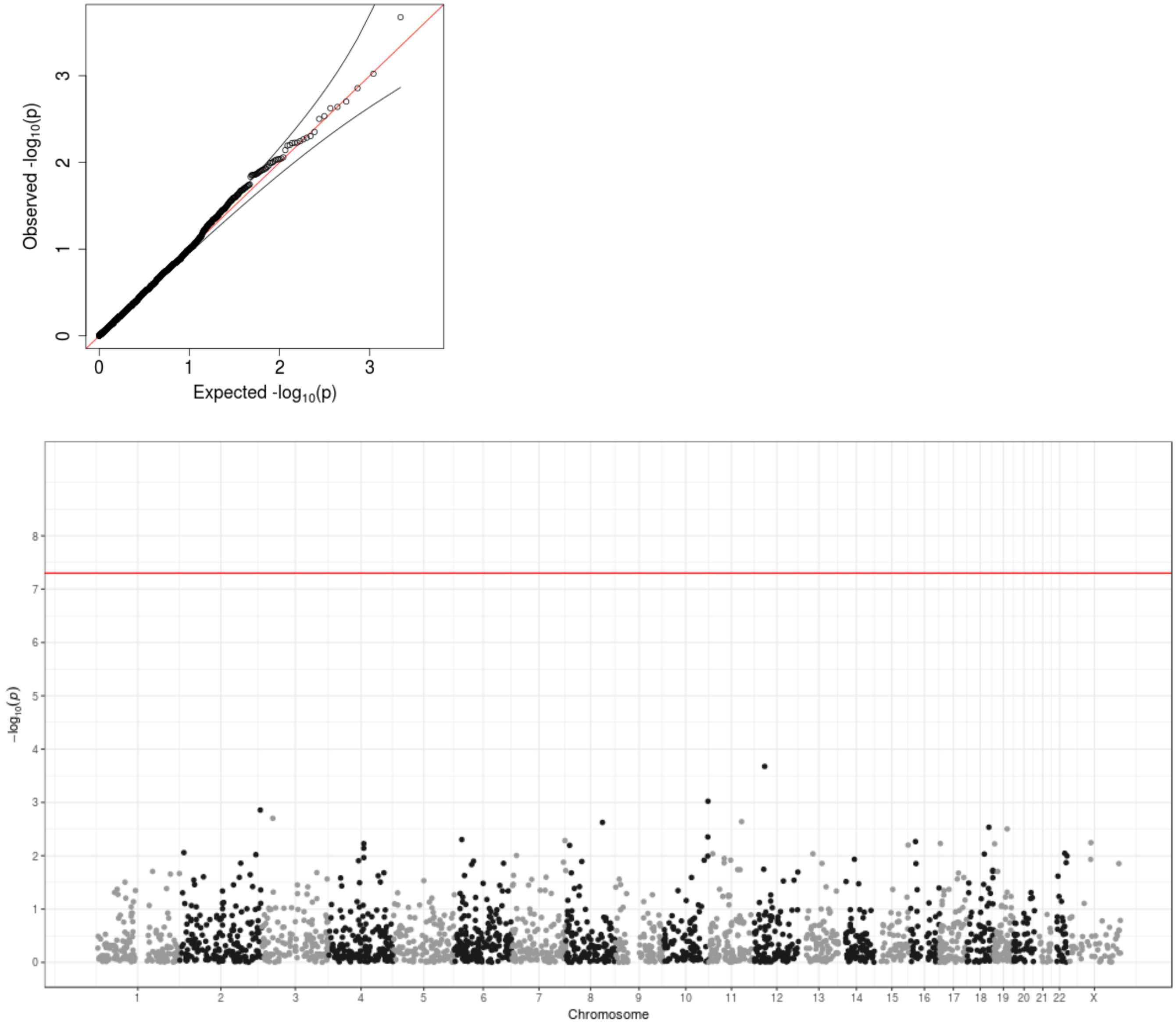

QQ and Manhattan plots for DEL (2,199 variants). We obtain a λ_{GC} of 1. A logistic regression model with additive genetic model (Plink --logistic) with empirically determined covariates (PC2 as covariate for chr1-22, PC2 and sex as covariates for chrX) was used to estimate association between single variants and schizophrenia. Statistical tests are two-sided. *P* values shown in the figures are the asymptotic *P* values from Plink outputs. To correct for multiple comparisons in the analysis of common variant association, we used the established genome-wide significance threshold of 5×10^{-8}.

Supplementary Figure 22: QQ and Manhattan plots for common DUP

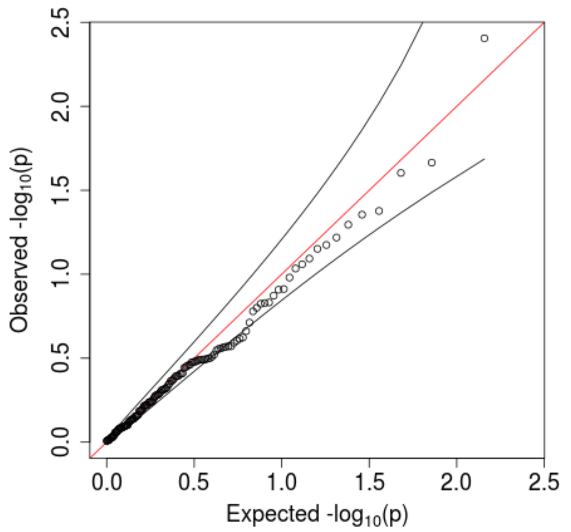

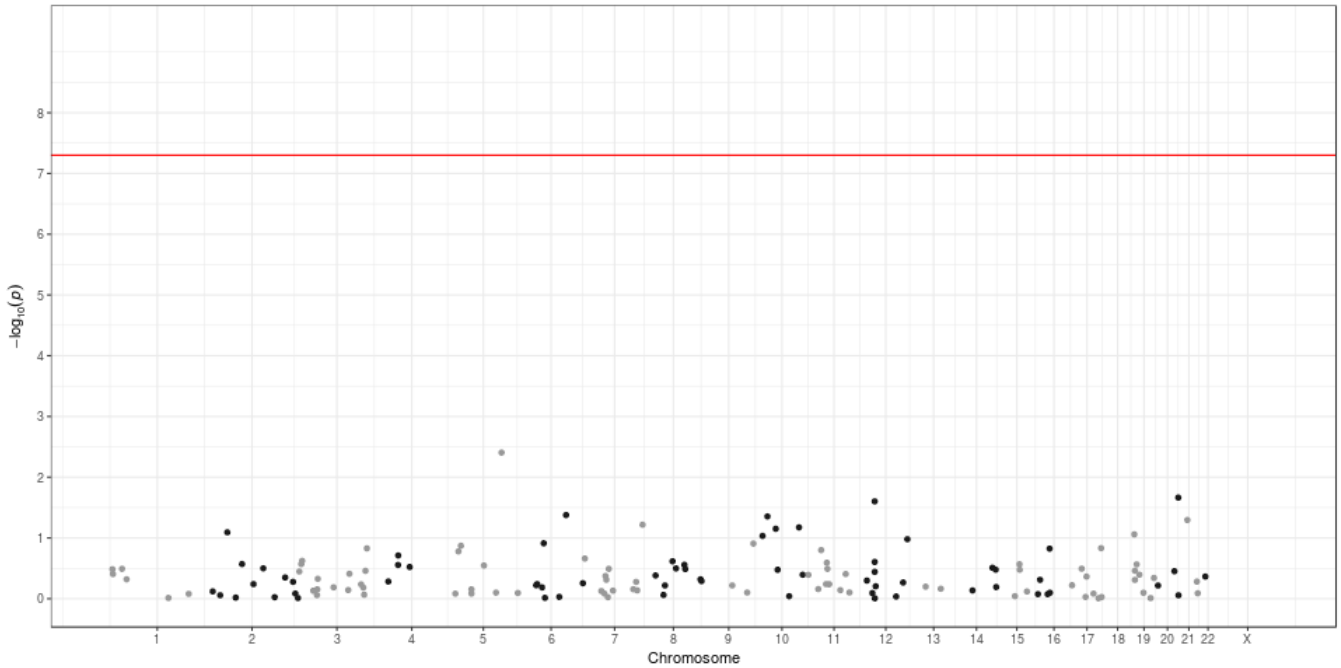

QQ and Manhattan plots for DUP (144 variants). We obtain a λ_{GC} of 1.05. A logistic regression model with additive genetic model (Plink --logistic) with empirically determined covariates (PC2 as covariate for chr1-22, PC2 and sex as covariates for chrX) was used to estimate association between single variants and schizophrenia. Statistical tests are two-sided. *P* values shown in the figures are the asymptotic *P* values from Plink outputs. To correct for multiple comparisons in the analysis of common variant association, we used the established genome-wide significance threshold of $5x10^{-8}$.

Supplementary Figure 23: QQ and Manhattan plots for common INV

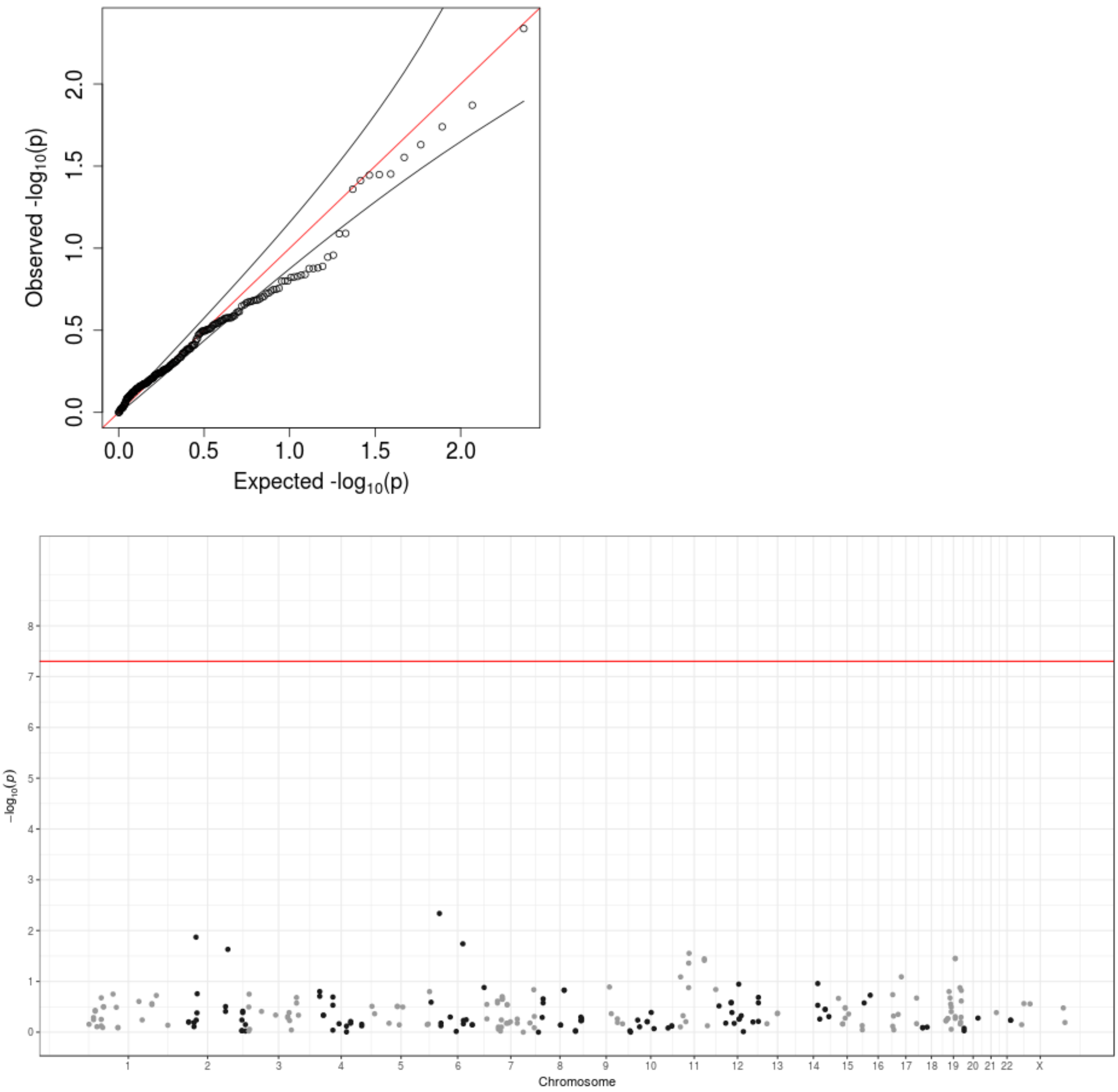

QQ and Manhattan plots for INV (234 variants). We obtain a λ_{GC} of 1. A logistic regression model with additive genetic model (Plink --logistic) with empirically determined covariates (PC2 as covariate for chr1-22, PC2 and sex as covariates for chrX) was used to estimate association between single variants and schizophrenia. Statistical tests are two-sided. *P* values shown in the figures are the asymptotic *P* values from Plink outputs. To correct for multiple comparisons in the analysis of common variant association, we used the established genome-wide significance threshold of $5x10^{-8}$.

Supplementary Figure 24: QQ and Manhattan plots for common ALU

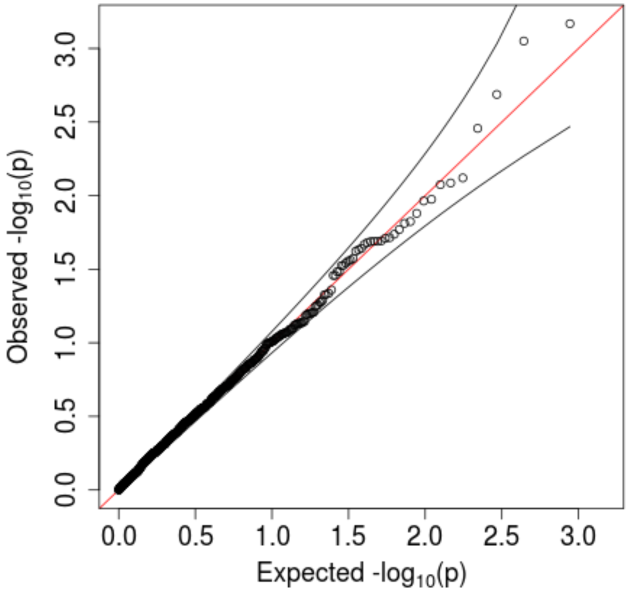

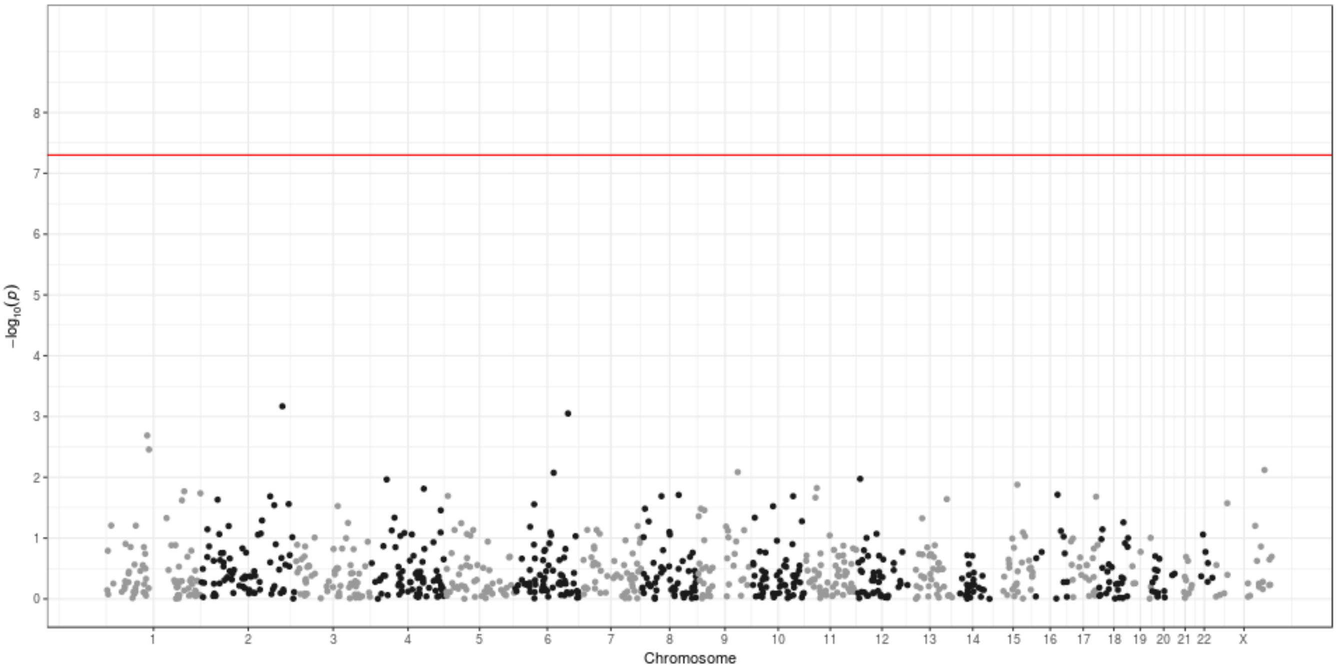

QQ and Manhattan plots for ALU (880 variants). We obtain a λ_{GC} of 1.05. A logistic regression model with additive genetic model (Plink --logistic) with empirically determined covariates (PC2 as covariate for chr1-22, PC2 and sex as covariates for chrX) was used to estimate association between single variants and schizophrenia. Statistical tests are two-sided. *P* values shown in the figures are the asymptotic *P* values from Plink outputs. To correct for multiple comparisons in the analysis of common variant association, we used the established genome-wide significance threshold of $5x10^{-8}$.

Supplementary Figure 25: QQ and Manhattan plots for common LINE1

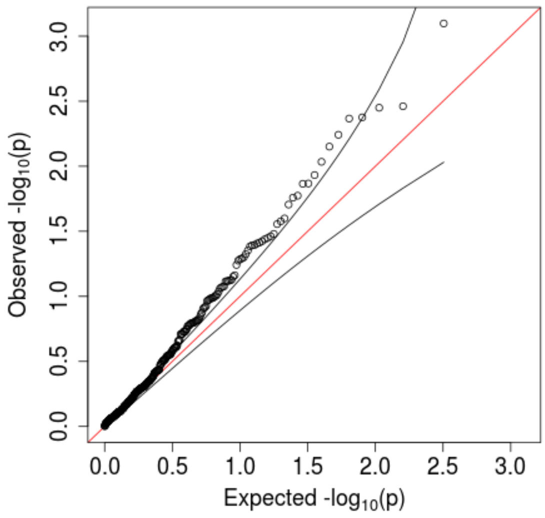

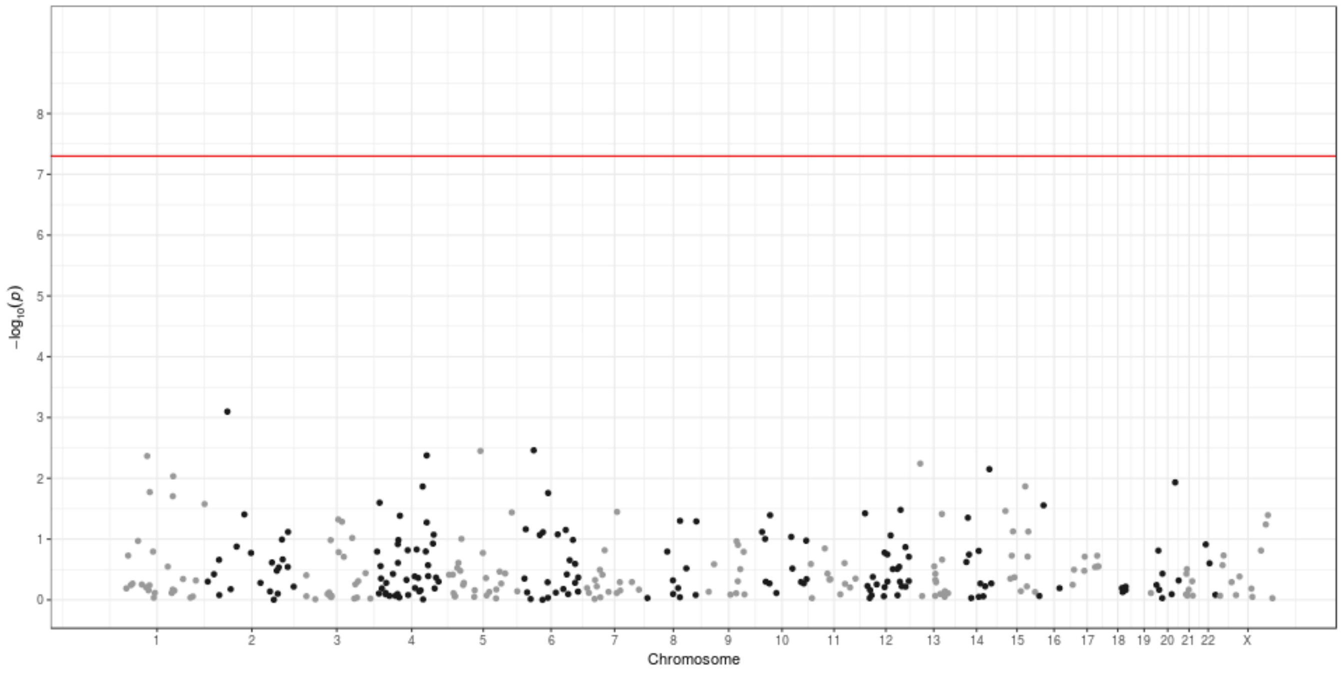

QQ and Manhattan plots for LINE1 (320 variants). We obtain a λ_{GC} of 1.12. A logistic regression model with additive genetic model (Plink --logistic) with empirically determined covariates ($\mathrm{PC2}$ as covariate for chr1-22, $\mathrm{PC2}$ and sex as covariates for chrX) was used to estimate association between single variants and schizophrenia. Statistical tests are two-sided. *P* values shown in the figures are the asymptotic *P* values from Plink outputs. To correct for multiple comparisons in the analysis of common variant association, we used the established genome-wide significance threshold of 5×10^{-8}.

Supplementary Figure 26: QQ and Manhattan plots for common SVA

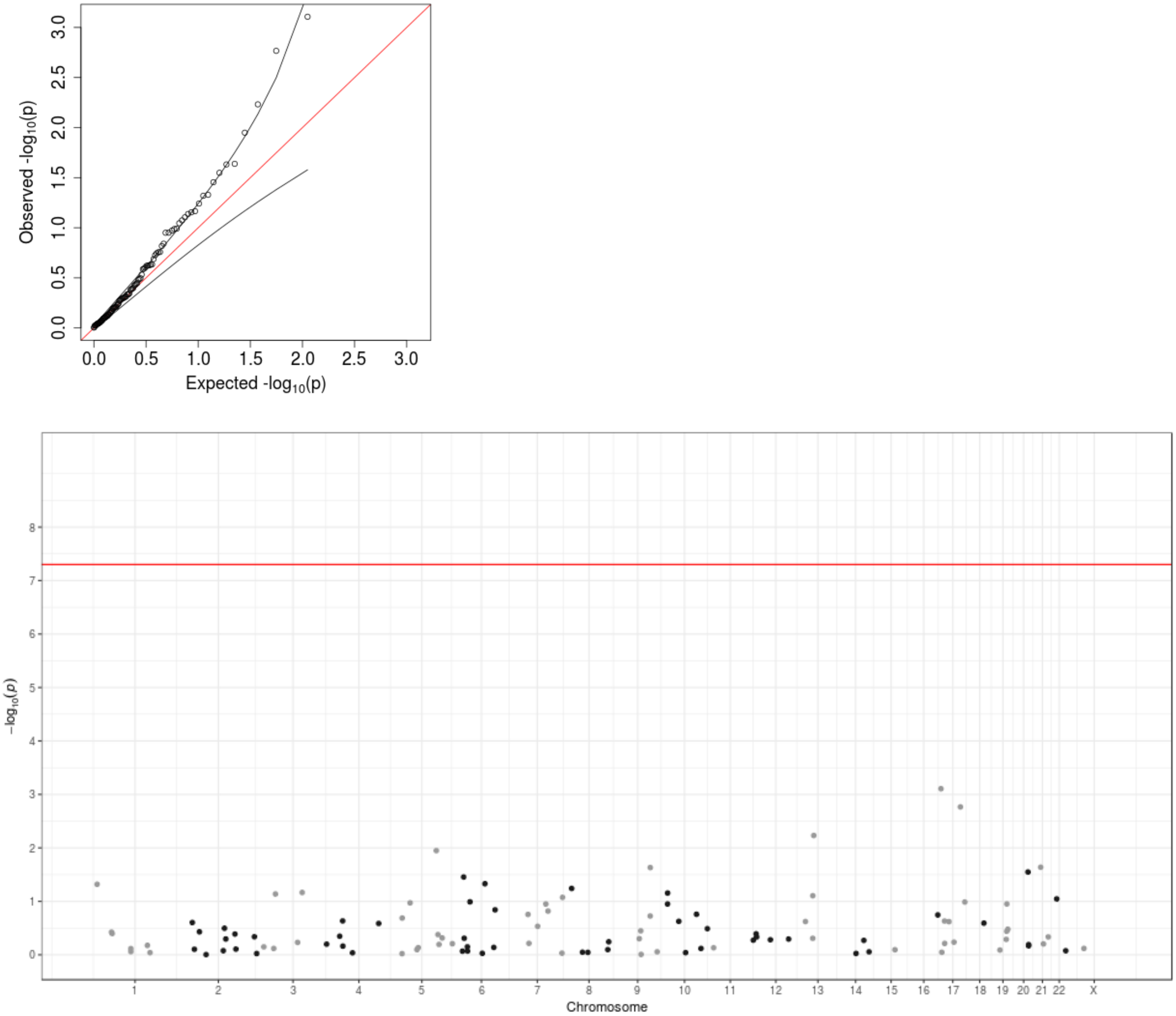

QQ and Manhattan plots for SVA (112 variants). We obtain a λ_{GC} of 1. A logistic regression model with additive genetic model (Plink --logistic) with empirically determined covariates (PC2 as covariate for chr1-22, PC2 and sex as covariates for chrX) was used to estimate association between single variants and schizophrenia. Statistical tests are two-sided. *P* values shown in the figures are the asymptotic *P* values from Plink outputs. To correct for multiple comparisons in the analysis of common variant association, we used the established genome-wide significance threshold of $5x10^{-8}$.

Supplementary Figure 27: Heritability estimation using WGS

Supplementary Figure **27a HRC-imputable**	Supplementary Figure **27b WGS**
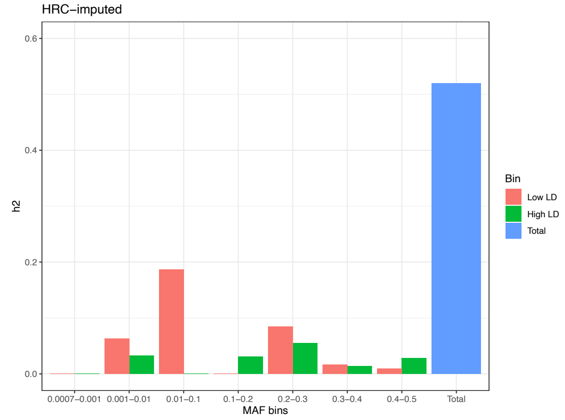	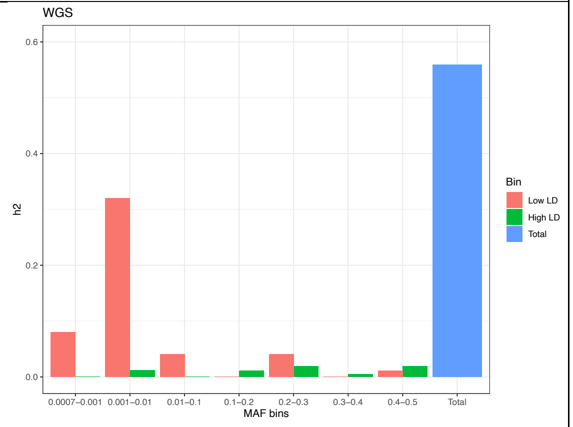

GREML-LDMS estimates stratified in 14 bins according to 7 MAF bins and 2 LD bins (0.0007 ~ 0.001, 0.001 ~ 0.01, 0.01 ~ 0.1, 0.1 ~ 0.2, 0.2 ~ 0.3, 0.3 ~ 0.4, 0.4 ~ 0.5) for MAF bins and LD < LD median, LD >= LD median for LD bins) with correction for 10 PCs (computed using HapMap3 SNPs) in 2,062 post-QC individuals.

(a) Estimates from HRC-imputable variants (8,498,854 variants). The number of variants in each of the 7 MAF bins (twice the number on each LD bin) is respectively, from lowest to highest MAF and boundary variants are included in consecutive bins: 378,120; 2,309,796; 2,932,222; 1,122,649; 750,758; 571,947; and 434,263.

(b) Estimates from WGS variants (17,364,971 variants). The number of variants in each of the 7 MAF bins (twice the number on each LD bin) is respectively, from lowest to highest MAF and boundary variants are included in consecutive bins: 2,697,651; 5,447,820; 4,122,379; 1,679,622; 1,267,561; 1,113,479; and 1,038,265.

Supplementary References

1. Reay, W.R. *et al.* Polygenic disruption of retinoid signalling in schizophrenia and a severe cognitive deficit subtype. *Mol Psychiatry* (2018).
2. Merico, D. *et al.* Whole-genome sequencing suggests schizophrenia risk mechanisms in humans with 22q11.2 deletion syndrome. *G3 (Bethesda)* **5**, 2453-61 (2015).
3. Khan, F.F. *et al.* Whole genome sequencing of 91 multiplex schizophrenia families reveals increased burden of rare, exonic copy number variation in schizophrenia probands and genetic heterogeneity. *Schizophr Res* (2018).
4. Tang, J. *et al.* Whole-genome sequencing of monozygotic twins discordant for schizophrenia indicates multiple genetic risk factors for schizophrenia. *J Genet Genomics* **44**, 295-306 (2017).
5. Castellani, C.A. *et al.* Post-zygotic genomic changes in glutamate and dopamine pathway genes may explain discordance of monozygotic twins for schizophrenia. *Clin Transl Med* **6**, 43 (2017).
6. Homann, O.R. *et al.* Whole-genome sequencing in multiplex families with psychoses reveals mutations in the SHANK2 and SMARCA1 genes segregating with illness. *Mol Psychiatry* **21**, 1690-1695 (2016).
7. Steinberg, S. *et al.* Truncating mutations in RBM12 are associated with psychosis. *Nat Genet* **49**, 1251-1254 (2017).
8. Chen, J. *et al.* A frameshift variant in the CHST9 gene identified by family-based whole genome sequencing is associated with schizophrenia in Chinese population. *Sci Rep* **9**, 12717 (2019).
9. Bundo, M. *et al.* Increased l1 retrotransposition in the neuronal genome in schizophrenia. *Neuron* **81**, 306-13 (2014).
10. Zhu, X. *et al.* Machine learning reveals bilateral distribution of somatic L1 insertions in human neurons and glia. *bioRxiv* (2019).
11. Purcell, S.M. *et al.* A polygenic burden of rare disruptive mutations in schizophrenia. *Nature* **506**, 185-90 (2014).
12. Genovese, G. *et al.* Increased burden of ultra-rare protein-altering variants among 4,877 individuals with schizophrenia. *Nat Neurosci* **19**, 1433-1441 (2016).
13. Ripke, S. *et al.* Genome-wide association analysis identifies 13 new risk loci for schizophrenia. *Nat Genet* **45**, 1150-9 (2013).
14. Szatkiewicz, J.P. *et al.* Copy number variation in schizophrenia in Sweden. *Mol Psychiatry* (2014).
15. McMurray, C.T. Mechanisms of trinucleotide repeat instability during human development. *Nat Rev Genet* **11**, 786-99 (2010).

16. La Spada, A.R., Wilson, E.M., Lubahn, D.B., Harding, A.E. & Fischbeck, K.H. Androgen receptor gene mutations in X-linked spinal and bulbar muscular atrophy. *Nature* **352**, 77-9 (1991).

17. Chong, S.S. *et al.* Gametic and somatic tissue-specific heterogeneity of the expanded SCA1 CAG repeat in spinocerebellar ataxia type 1. *Nat Genet* **10**, 344-50 (1995).

18. Imbert, G. *et al.* Cloning of the gene for spinocerebellar ataxia 2 reveals a locus with high sensitivity to expanded CAG/glutamine repeats. *Nat Genet* **14**, 285-91 (1996).

19. Gijselinck, I. *et al.* The C9orf72 repeat size correlates with onset age of disease, DNA methylation and transcriptional downregulation of the promoter. *Mol Psychiatry* **21**, 1112-24 (2016).

20. Nordin, A. *et al.* Extensive size variability of the GGGGCC expansion in C9orf72 in both neuronal and non-neuronal tissues in 18 patients with ALS or FTD. *Hum Mol Genet* **24**, 3133-42 (2015).

21. DeJesus-Hernandez, M. *et al.* Expanded GGGGCC hexanucleotide repeat in noncoding region of C9ORF72 causes chromosome 9p-linked FTD and ALS. *Neuron* **72**, 245-56 (2011).

22. Lalioti, M.D. *et al.* Identification of mutations in cystatin B, the gene responsible for the Unverricht-Lundborg type of progressive myoclonus epilepsy (EPM1). *Am J Hum Genet* **60**, 342-51 (1997).

23. Lalioti, M.D. *et al.* A PCR amplification method reveals instability of the dodecamer repeat in progressive myoclonus epilepsy (EPM1) and no correlation between the size of the repeat and age at onset. *Am J Hum Genet* **62**, 842-7 (1998).

24. Myers, R.H. Huntington's disease genetics. *NeuroRx* **1**, 255-62 (2004).

25. Malhotra, D. & Sebat, J. CNVs: Harbingers of a rare variant revolution in psychiatric genetics. *Cell* **148**, 1223-41 (2012).

26. Marshall, C.R. *et al.* Contribution of copy number variants to schizophrenia from a genome-wide study of 41,321 subjects. *Nat Genet* **49**, 27-35 (2017).

27. The Genomes Project, C. *et al.* A global reference for human genetic variation. *Nature* **526**, 68 (2015).
