# Supplementary material for: Increased burden of ultra-rare structural variants localizing to boundaries of topologically associated domains in schizophrenia
Source: Nat Commun. 2020 Apr 15;11:1842. doi: 10.1038/s41467-020-15707-w (PMC7160146; doi:10.1038/s41467-020-15707-w)
Supplement: Supplementary file 3 — Reporting Summary [file 41467_2020_15707_MOESM3_ESM.pdf]

nature research

Corresponding author(s): Jin Szatkiewicz

Last updated by author(s): 03/16/2020

Reporting Summary

Nature Research wishes to improve the reproducibility of the work that we publish. This form provides structure for consistency and transparency in reporting. For further information on Nature Research policies, see Authors & Referees and the Editorial Policy Checklist .

Statistics

For all statistical analyses, confirm that the following items are present in the figure legend, table legend, main text, or Methods section.

n/a	Confirmed	
☐	☒	The exact sample size (*n*) for each experimental group/condition, given as a discrete number and unit of measurement
☐	☒	A statement on whether measurements were taken from distinct samples or whether the same sample was measured repeatedly
☐	☒	The statistical test(s) used AND whether they are one- or two-sided<br>*Only common tests should be described solely by name; describe more complex techniques in the Methods section.*
☐	☒	A description of all covariates tested
☐	☒	A description of any assumptions or corrections, such as tests of normality and adjustment for multiple comparisons
☐	☒	A full description of the statistical parameters including central tendency (e.g. means) or other basic estimates (e.g. regression coefficient) AND variation (e.g. standard deviation) or associated estimates of uncertainty (e.g. confidence intervals)
☐	☒	For null hypothesis testing, the test statistic (e.g. *F*, *t*, *r*) with confidence intervals, effect sizes, degrees of freedom and *P* value noted<br>*Give P values as exact values whenever suitable.*
☒	☐	For Bayesian analysis, information on the choice of priors and Markov chain Monte Carlo settings
☒	☐	For hierarchical and complex designs, identification of the appropriate level for tests and full reporting of outcomes
☐	☒	Estimates of effect sizes (e.g. Cohen's *d*, Pearson's *r*), indicating how they were calculated

Our web collection on statistics for biologists contains articles on many of the points above.

Software and code

Policy information about availability of computer code

Data collection	No other direct software used for data collection. JMP (v11, https://www.jmp.com/) was used to combine information and extract cases for whole genome sequencing.
Data analysis	Analysis software used in this study include the following: HiSeq Control Software 3.3.39/RTA 2.7.1; Piper (v1.4.0, http://doi.org/10.5281/zenodo.154586); bwa (v0.7.12, http://bio-bwa.sourceforge.net/); SAMtools (v0.1.19, http://samtools.sourceforge.net/); Picard (v1.120, https://broadinstitute.github.io/picard/); qualimap (v2.2, http://qualimap.bioinfo.cipf.es/); FastQC (v0.11.4, https://www.bioinformatics.babraham.ac.uk/projects/fastqc/); BEDTools (v2.28.0, https://bedtools.readthedocs.io/en/latest/); GATK (v3.3, https://software.broadinstitute.org/gatk/); PLINK (v1.9, https://www.cog-genomics.org/plink/1.9/); PLINK (v1.07, http://zzz.bwh.harvard.edu/plink/); ExpansionHunter (v2.5.5, https://github.com/Illumina/ExpansionHunter); Delly (v0.7.7, https://github.com/dellytools/delly); MELT (v2, http://melt.igs.umaryland.edu/manual.php); VEP (v91, https://github.com/Ensembl/ensembl-vep); vcfanno (v0.2.9, https://github.com/brentp/vcfanno); AnnotSV (v1.1.1, https://lbgi.fr/AnnotSV/); VCFscreen (v0.1, https://github.com/Halvee/VCFScreen); R (v3.2.2., https://www.r-project.org); R/gap package (https://github.com/jinghuazhao/R); GCTA (v1.26.0, v1.92.3beta, https://cnsgenomics.com/software/gcta/#Overview); JMP (v11, https://www.jmp.com/); AbCD Calculator (https://yunliweb.its.unc.edu/abcd_web/AbCD.php). Python code “concordance.py” and other relevant codes are posted at https://github.com/jinszatkiewicz/swsczwgs.<br>All software and versions are stated throughout Methods.

For manuscripts utilizing custom algorithms or software that are central to the research but not yet described in published literature, software must be made available to editors/reviewers. We strongly encourage code deposition in a community repository (e.g. GitHub). See the Nature Research guidelines for submitting code & software for further information.

Data

Policy information about availability of data

All manuscripts must include a data availability statement. This statement should provide the following information, where applicable:

- Accession codes, unique identifiers, or web links for publicly available datasets
- A list of figures that have associated raw data
- A description of any restrictions on data availability

Summary statistics from single-variant association analysis in this study can be downloaded from Psychiatric Genomics Consortium's website at https://www.med.unc.edu/pgc/download-results/causal-variants-within-scz/. All other summary statistics and supporting data are available in Supplementary Information. Due to recent changes in Swedish and European Union regulations regarding genetic data, we are unable to deposit individual-level data into controlled-access repositories like dbGaP. Collaborative analyses are possible and can be pursued by contacting the authors.

Field-specific reporting

Please select the one below that is the best fit for your research. If you are not sure, read the appropriate sections before making your selection.

☒ Life sciences ☐ Behavioural & social sciences ☐ Ecological, evolutionary & environmental sciences

For a reference copy of the document with all sections, see nature.com/documents/nr-reporting-summary-flat.pdf

Life sciences study design

All studies must disclose on these points even when the disclosure is negative.

Sample size	Prior studies demonstrated that power to implicate schizophrenia risk loci is only sufficient with a large case/control cohort on the order of > 10,000 individuals. We have not reached this desired sample size but the sample size in the current study was chosen to balance the statistical power and the financial and computational burden inherent to high-coverage whole genome sequencing (WGS). The sample size in this study was chosen because: (1) To determine statistical power and estimate sample size needed for variant detection, we used the AbCD calculator and performed simulations assuming 30x WGS, EUR ancestry, and various sample sizes. We found, when assuming ~2000 EUR individuals and 30x WGS, we have > 99% power to detect variants at MAF > 0.001. (2) For power calculation for association analysis given the sample, we used the R/gap package. We assumed an additive model, lifetime risk of schizophrenia of 1%, type I error level of 5x10^-8, or 1x10^-5; and we computed the minimal detectable genotypic relative risk to achieve 20%, 80% power over a range of frequency of risk alleles in the population (Supplementary Figure 6). For URV burden and given 2,098 individuals, we had ≥ 80% power to detect an association when the aggregated minor allele count is 20 (i.e., aggregated MAF=0.01) and the genotypic relative risk was ≥ 4.9, assuming a type I error level of 1x10^-5. For common variant association and given 2098 individuals, we had ≥80% power to detect risk variants with MAF=0.25 and genetic relative risks ≥ 2.0, assuming a type I error level of 5x10^-8.
Data exclusions	We used standard quality control procedures for variant discovery and for genetic association studies to retain high quality samples and variants for data analysis. These procedures including the exclusions and their rationale are described explicitly in Online Methods and summarized here.<br>For subject quality control, we excluded 9 subjects for failed sequencing quality metrics (1 case excluded), sex mismatch (1 control excluded), sex chromosomal abnormality (2 cases with XXY excluded), and one of any pair of subjects with high relatedness $\hat{\pi}$ > 0.2 (5 controls excluded). These procedures resulted in a final sample size of 2,098 subjects (1,162 schizophrenia cases and 936 controls), all of whom had SNV/indel missing rate per sample < 0.01 and heterozygosity rate < 0.1. In selection of the schizophrenia cases, we excluded carriers of known large pathogenic CNVs and abnormally high total number of CNVs using SNP arrays. These QC procedures were done using PLINK (v1.9).<br>For SNV/indels quality control, we removed variants if missing rate per variant > 0.01 (before sample removal) and applied genotype QC by setting low quality genotypes with DP<10 or GQ<20 as missing. We then removed variants that were: monomorphic, missing rate per variant > 0.02 (after genotype QC and sample removal), missing rate per variant difference in cases and controls > 0.02 or P <0.005, Hardy-Weinberg equilibrium FDR < 1x10^-6 (controls) or < 1x10^-10 (cases). After QC, we extracted variants with minor allele frequency (MAF) ≥ 0.01 for common variant association analysis and the remaining for rare variant aggregated association analysis. These QC procedures were done using PLINK (v1.9).<br>For structural variants quality control, we removed variants if they overlapped by more than 66% with large genome gaps (e.g., centromeres), segmental duplications, or regions subject to somatic V(D)J recombination in white blood cells, with the logic that these variant calls are likely artifactual. Finally, we extracted variants with MAF ≥0.01 for common variant association analysis and the remaining for rare variant aggregated association analysis. These QC procedures were done using PLINK (v1.07).
Replication	We did not carry out a replication study in an independent sample. However, we took multiple measures to verify the reproducibility of the experimental findings. (1) We evaluated and confirmed high genotype accuracy of the variants discovered in this study by comparing to prior technologies (SNP array, exome sequencing) applied to the same samples. (2) We replicated key prior reported excess in schizophrenia of LoF URVs in genes that are putatively LoF intolerant as well as rare deletions genome-wide. (3) We evaluated and verified the validity of structural variants that affected TAD boundaries in schizophrenia cases by comparing to prior technologies and inspecting read alignments.
Randomization	No randomization was carried out. This study is a large-scale genetic study of unrelated individuals. Randomness is achieved from the nature that alleles are randomly passed from parents to offspring during meiosis.
Blinding	No blinding was carried out. Researchers were not blinded to case/control status. Researchers were blinded to the genetic makeup of the study subjects by the nature of the study, as no one would know beforehand the genotypes of the cases and controls.

Reporting for specific materials, systems and methods

We require information from authors about some types of materials, experimental systems and methods used in many studies. Here, indicate whether each material, system or method listed is relevant to your study. If you are not sure if a list item applies to your research, read the appropriate section before selecting a response.

Materials & experimental systems

n/a	Involved in the study
☒	☐ Antibodies
☒	☐ Eukaryotic cell lines
☒	☐ Palaeontology
☒	☐ Animals and other organisms
☐	☒ Human research participants
☒	☐ Clinical data

Methods

n/a	Involved in the study
☒	☐ ChIP-seq
☒	☐ Flow cytometry
☒	☐ MRI-based neuroimaging

Human research participants

Policy information about studies involving human research participants

Population characteristics

Only de-identified samples with case/control status and sex are provided to the team of this study. There are 1162 Swedish schizophrenia cases and 936 ancestry-matched population controls from Sweden, of which 1230 are male and 868 are female. For schizophrenia cases, all are aged >=18. For SweGen controls, phenotype data including age was not allowed in the SweGen project in order to make a less restrictive access policy possible.

Recruitment

All schizophrenia cases included this study are from the Swedish Schizophrenia Study (S3). S3 cases were identified via the Swedish Hospital Discharge Register that captures >99% of all inpatient hospitalizations in Sweden.The register is complete from 1987 and augmented by psychiatric data from 1973-86. The sampling frame is thus population-based and covers all hospital-treated patients. The Hospital Discharge Register contains dates and ICD discharge diagnoses for each hospitalization, and captures the clinical diagnosis made by attending physicians. Case inclusion criteria: ≥2 hospitalizations with a discharge diagnosis of schizophrenia or schizoaffective disorder, both parents born in Scandinavia, and age ≥18 years. Case exclusion criteria: hospital register diagnosis of any medical or psychiatric disorder mitigating a confident diagnosis of schizophrenia as determined by expert review, and included removal of 3.4% of eligible cases due to the primacy of another psychiatric disorder (0.9%) or a general medical condition (0.3%) or uncertainties in the Hospital Discharge Register (e.g., contiguous admissions with brief total duration, 2.2%). Potential cases were contacted directly via an introductory letter followed by a telephone call. If they agreed, a research nurse met them at a psychiatric treatment facility or in their home, obtained written informed consent, obtained a blood sample, and conducted a brief interview about other medical conditions in a lifetime. From the S3 schizophrenia cases (more than 5,000), we selected 1,165 cases for whole genome sequencing (WGS) in the current study. Our main goal in selection was typical Swedish ancestry and clear schizophrenia caseness. Cases carrying known pathogenic copy number variants (CNVs) (e.g. 22q11del, 16p11dup) were not selected as a primary question of this study is to evaluate the contribution of novel loci on schizophrenia risk. DNA was extracted from peripheral blood samples. Specifically, our selection procedures required the following case inclusion criteria to be met: (1) have high-quality/sufficient DNA that satisfied all criteria: concentration ≥ 80 μg/ml, volume ≥150 μl, and purity ratio 1.7-2.2; (2) used in GWA study; (3) have typical Swedish ancestry defined by the first two PCs; (4) do not carry known large pathogenic CNVs and are not outliers for total number of CNVs; (5) have stringent evidence of schizophrenia that satisfied all criteria: >8 inpatient or outpatient psychiatric treatment contacts for schizophrenia or schizoaffective disorder, ≥30 inpatient days for schizophrenia, ≥5 redeemed prescriptions for antipsychotics, and few or no treatment contacts for bipolar disorder.

All control subjects included this study are from the SweGen project, a population-based high-quality genetic variant dataset for the Swedish population. One of the aims of SweGen is to enable WGS association studies for national patient cohorts studies in Sweden, by providing data on well-matched national controls selected on the basis of the genetic structure of the Swedish population. Detailed description of the SweGen subjects are available elsewhere 48 and are briefly summarized here. SweGen project included a total 1,000 individuals, of which 942 individuals were selected from The Swedish Twin Registry (STR) 49 and 58 from The Northern Swedish Population Health Study (NSPHS)50. Both STR and NSPHS are population-based collections and were approved by local ethics committees. STR is a national registry of Swedish born twins established in the 1960s and, at present, holds information on 85,000 twin pairs. 11,000 individuals from the STR (one per monozygous twin pairs) participated in TwinGene and had existing SNP array genotyping. The Twingene study is a nation-wide and population-based study of Swedish born twins agreeing to participate. The TwinGene sample collection represents the Swedish geographic population density distribution. Based on principal component analysis (PCA), 942 unrelated individuals were selected from TwinGene participants for whole genome sequencing, mirroring the density distribution. Given the selected 1,000 subjects that constitutes SweGen, a PCA using genotypes from high-density SNP arrays was performed and confirmed that the SweGen control cohort captured the diversity in the country. Furthermore, since STR and NSPHS are already established national sample collections that do not reflect recent migration patterns, the SweGen control cohort is likely to reflect the genetic structure of Swedish individuals that have been present in Sweden for at least one generation. From the SweGen subjects, we selected the 942 STR/TwinGene individuals as controls in this study because of their matched ancestry with selected schizophrenia cases. Phenotype data was not allowed in the SweGen project in order to make a less restrictive access policy possible. Consequently, we were unable to screen for the presence of individuals with schizophrenia. However, we estimate that at most 1 control individual may carry a schizophrenia diagnosis (given the estimated schizophrenia prevalence of 0.0009 in the full STR/TwinGene project of 11,000 individuals). Misclassification of a single control subject will not likely affect the results or the power of the study.

Ethics oversight	University of North Carolina (Institutional Review Boards); Karolinska Institutet (Regionala Etikprövningsnämnden, Stockholm); University of Uppsala (Regionala Etikprövningsnämnden, Uppsala).

Note that full information on the approval of the study protocol must also be provided in the manuscript.
